# Alleviation of Microglial Activation Induced by p38 MAPK/MK2/PGE_2_ Axis by Capsaicin: Potential Involvement of other than TRPV1 Mechanism/s

**DOI:** 10.1038/s41598-017-00225-5

**Published:** 2017-03-08

**Authors:** Harsharan S. Bhatia, Nora Roelofs, Eduardo Muñoz, Bernd L. Fiebich

**Affiliations:** 1 Department of Psychiatry and Psychotherapy, University of Freiburg Medical School, Hauptstrasse 5, D-79104 Freiburg, Germany; 2 VivaCell Biotechnology GmbH, Ferdinand-Porsche-Strasse 5, D-79211 Denzlingen, Germany; 3 Maimonides Biomedical Research Institute of Córdoba, Reina Sofía University Hospital, Department of Cell Biology, Physiology and Immunology, University of Córdoba, Avda Menéndez Pidal s/n., 14004 Córdoba, Spain; 4 VivaCell Biotechnology España, Parque Científico Tecnológico Rabanales 21, 14014 Córdoba, Spain

## Abstract

Exaggerated inflammatory responses in microglia represent one of the major risk factors for various central nervous system’s (CNS) associated pathologies. Release of excessive inflammatory mediators such as prostaglandins and cytokines are the hallmark of hyper-activated microglia. Here we have investigated the hitherto unknown effects of capsaicin (cap) - a transient receptor potential vanilloid 1 (TRPV1) agonist- in murine primary microglia, organotypic hippocampal slice cultures (OHSCs) and human primary monocytes. Results demonstrate that cap (0.1–25 µM) significantly (p < 0.05) inhibited the release of prostaglandin E_2_ (PGE_2_)_,_ 8-iso-PGF_2α,_ and differentially regulated the levels of cytokines (TNF-α, IL-6 & IL-1β). Pharmacological blockade (via capsazepine & SB366791) and genetic deficiency of TRPV1 (TRPV1^−/−^) did not prevent cap-mediated suppression of PGE_2_ in activated microglia and OHSCs. Inhibition of PGE_2_ was partially dependent on the reduced levels of PGE_2_ synthesising enzymes, COX-2 and mPGES-1. To evaluate potential molecular targets, we discovered that cap significantly suppressed the activation of p38 MAPK and MAPKAPK2 (MK2). Altogether, we demonstrate that cap alleviates excessive inflammatory events by targeting the PGE_2_ pathway in *in vitro* and *ex vivo* immune cell models. These findings have broad relevance in understanding and paving new avenues for ongoing TRPV1 based drug therapies in neuroinflammatory-associated diseases.

## Introduction

The immune system plays an indispensable role in the maintenance of tissue homeostasis in response to infection and pathological insults. Microglia are the major resident immunocompetent cells in the brain, where they constantly survey the microenvironment in order to sustain homeostatic milieu^1, 2^. Under physiological conditions, microglia exhibit a ramified or deactivated phenotype that is associated with the production of various anti-inflammatory factors^1, 3, 4^. Infections, traumatic injury, ischemia, neurodegenerative diseases or any altered neuronal activity indicating a potential threat to central nervous system (CNS) can evoke profound changes in microglial morphology and function^5–8^. Sustained inflammation or failure in normal resolution mechanisms in microglia further leads to cellular damage. Under such conditions, microglia are known to release a variety of cytotoxic mediators, such as pro-inflammatory cytokines including tumour necrosis factor-alpha (TNF-α), interleukin (IL)-6 and IL-1β, reactive oxygen species, adenosine triphosphate (ATP), nitric oxide (NO), arachidonic acid (AA) derivatives, most importantly prostaglandin E_2_ (PGE_2_)^9–13^. Excessive release of PGE_2_ and cytokines during chronic neuroinflammation further exerts their toxic effects on neighbouring healthy neurons, and result in a vicious self-perpetuating cycle. Previously, dysregulation of PGE_2_ and its synthesizing enzymes were reported in a variety of CNS related pathologies including cerebral ischemia, psychiatric disorders and neurodegenerative diseases^14–17^.

The cyclooxygenases (COX) and prostaglandin (PG) E synthase (PGESs) enzymes catalyse synthesis of PGE_2_. The cyclooxygenases exist in two forms, constitutively expressed cyclooxygenase-1 (COX-1) and the inducible form cyclooxygenase-2 (COX-2). In our previous findings, we demonstrated that COX-2 can be overexpressed by the bacterial cell wall component, lipopolysaccharide (LPS), in cultured microglia^18^. Three forms of PGESs regulate the final step in the synthesis of PGE_2_. Among them, mPGES-1 is an inducible form, and we showed earlier that its expression is upregulated during microglial activation^19, 20^. COX-2 and mPGES-1 are both regulated at transcriptional levels and both enzymes are important in the synthesis of PGE_2_ during inflammation^21^. These enzymes are regulated by a variety of intracellular signalling molecules including nuclear factor-kappa B (NF-κB) and mitogen activated protein kinases (MAPK). In particular, previous studies demonstrate that p38 MAPK, and its downstream substrate mitogen-activated protein kinase-activated protein kinase-2 (MAPKAPK2 or MK2), plays paramount role in chronic inflammatory associated diseases, including neurodegenerative diseases^22–25^.

A growing body of evidence points to the role of ion channels on monocytes and microglia/brain macrophages in health and disease^26, 27^. Among others, the transient receptor potential vanilloid 1 (TRPV1) has recently gained a great deal of attention. TRPV1 is a nonselective cation channel classically known to be involved in the detection and transduction of nociceptive stimuli. Currently, modulators (either agonists or antagonists) of TRPV1 are being developed at pace to combat pain and inflammation-associated pathologies^28–30^. TRPV1 is primarily expressed in somatosensory neurons and is opened by capsaicin, heat reception (≥43 °C), protons and endovanilloids^31–33^.

**Figure 1:**
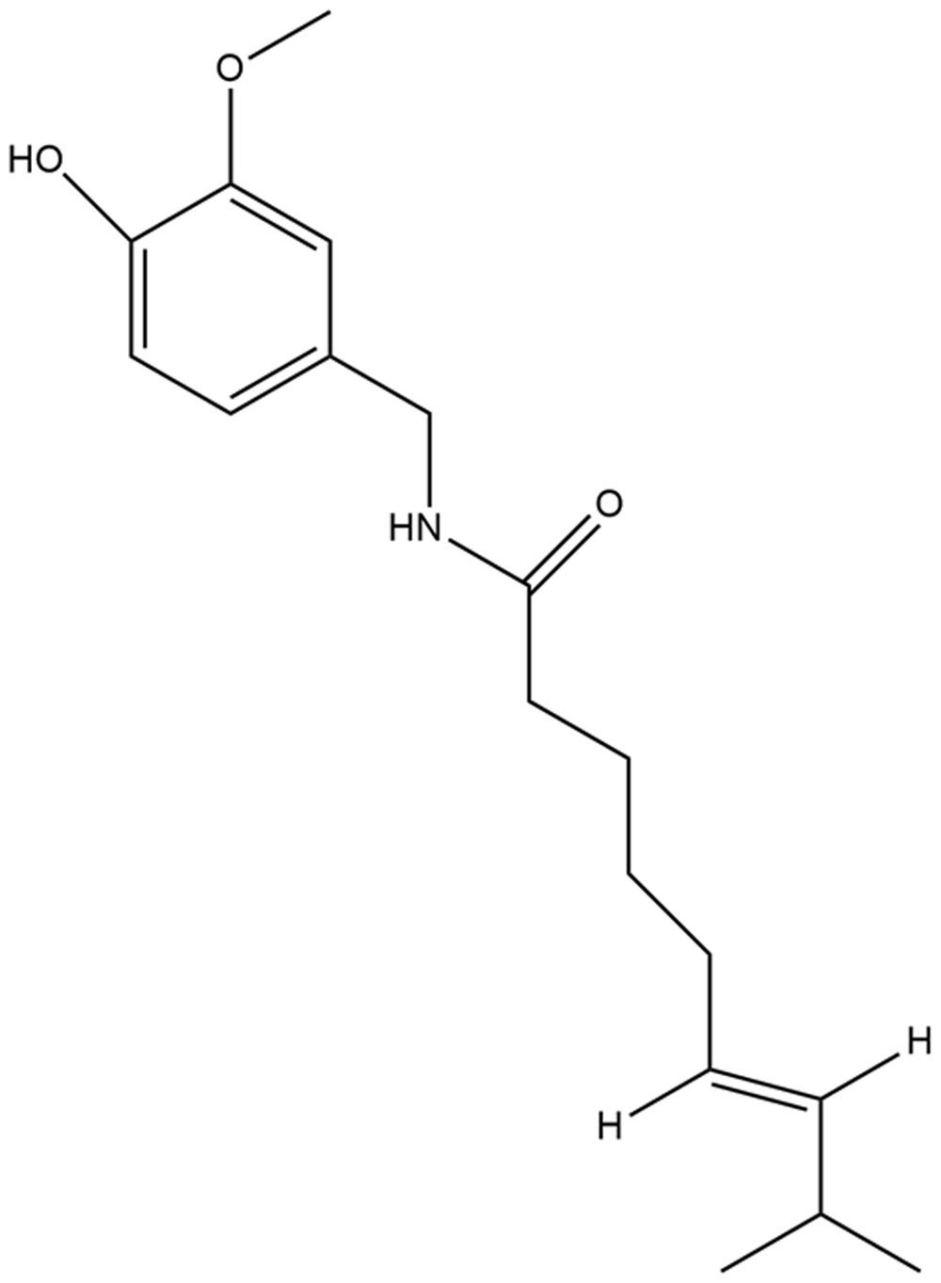
The molecular structure of capsaicin (C_18_H_27_NO_3_).

Capsaicin (*trans*-8-methyl-*N*-vanillyl-6-nonenamide) is a naturally occurring alkaloid derived from plants belonging to genus *capsicum*. The characteristic hot and spicy flavor of chili peppers is due to the presence of capsaicin (cap). Cap has a benzene ring and a long hydrophobic carbon tail with a polar amide group (Fig. 1) with the molecular formula C_18_H_27_NO_3_. Cap has been shown to exhibit variety of health promoting effects in various conditions, including obesity, diabetes, cardiovascular conditions, cancer, airway diseases, itch, and gastric disorders^30^. For further readings, we suggest two excellent reviews regarding the bioavailability, pharmacokinetics, molecular targets and beneficial effects of cap both in rodents and in humans^34, 35^. Understanding the actions of cap led to the discovery its receptor, TRPV1.

TRPV1 is not only expressed by neurons, but also by immune cells, including monocytes^36^ and macrophages^37^. Some reports suggest that microglia also express functional TRPV1, and its activation promotes cell migration and phagocytosis^38, 39^, while others have suggested its contribution to microglial cell death and oxidative stress^40, 41^. However, to date it is unknown whether cap and its putative receptor TRPV1 play any role in reducing excessive microglial activation in the context of membrane bound AA metabolism- a critical event in the progression of neuroinflammation. In particular, interference of cap on the p38 MAPK/MK2/PGE_2_ axis in activated microglia has never been explored. Therefore, the present study demonstrates a novel target of cap, independent of TRPV1, on the arachidonic acid pathway by using *in vitro* and *ex vivo* immune cell and tissue models.

## Results

### Suppression of PGE_2_ release and free radical formation (8-iso-PGF_2α_) by capsaicin in activated microglia without affecting the viability of cells

**Figure 2:**
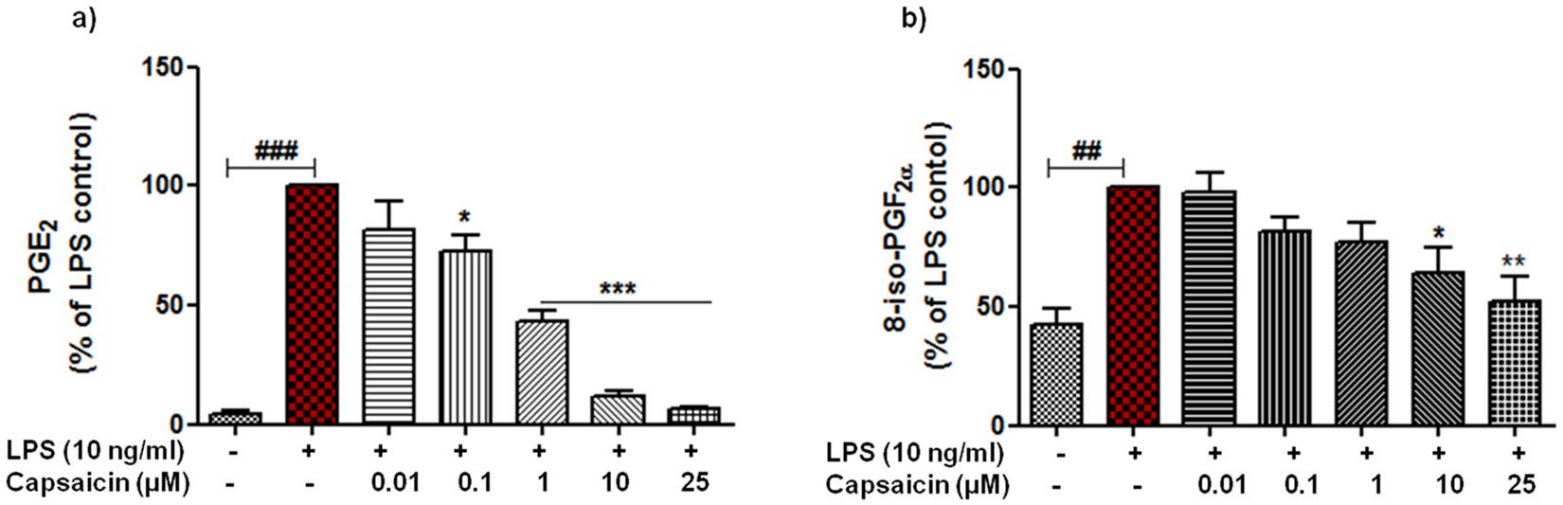
Capsaicin (cap) inhibits the release of prostaglandin E_2_ (PGE_2_) and 8-isoprostane (8-iso-PGF_2α_) in lipopolysaccharide (LPS)-activated microglia. Microglia were pre-treated with cap (0.01–25 µM) for 30 min, afterwards cells were incubated with or without LPS (10 ng/ml) for the next 24 h. At the end of incubation, cell supernatants were collected and release of PGE_2_ (**a**) and 8-iso-PGF_2α_ (**b**) were measured by enzyme immune assay (EIA). Statistical analysis was carried out by using one way ANOVA with *post hoc* student Newman-Keuls test (multiple comparisons). Results are expressed as means ± SE of at least 3–5 independent experiments. Significant differences were set as *p < 0.05; **p < 0.01; ***p < 0.001 compared with LPS (10 ng/ml) activated cells; ^##^p < 0.01; ^###^p < 0.001 compared with control cells.

To investigate whether cap exerts anti-inflammatory effects, microglia cells were pre-incubated with cap for 30 min and then stimulated with or without LPS (10 ng/ml) for given time points. As a result, we observed a marked increase in the production of PGE_2_, 8-iso-PGF_2α_ (Fig. 2a,b), TNF-α, IL-6, IL-1β and iNOS (see Supplementary Fig. S1) when stimulated with LPS as compared with unstimulated cells. Treatment with cap prior to stimulation with LPS resulted in significant decrease of PGE_2_ release without substantial effects on other inflammatory mediators when compared with LPS (considered as 100%). Significant reduction in the levels of PGE_2_ were evident starting from the concentration (conc.) of 0.1 µM (mean 72.40 ± 6.72%, p < 0.05, n = 5) and pronounced decrease was observed at the conc. of 25 µM (mean 6.60 ± 0.50%, p < 0.001) as shown in Fig. 2a. Previous studies showed the antioxidant properties of cap in variety of study models^42^. Therefore, we also speculated that cap might exert its anti-oxidative effects in activated microglia. To this end, we studied the effects of cap on the formation of free radicals in LPS activated microglia. Measurement of 8-iso-PGF_2α_ release is taken as a sensitive marker to assess free radical formation^43^ and we have previously shown that LPS significantly increases the levels of 8-iso-PGF_2α_ in primary microglia^21, 44^. Indeed, here we also observed that LPS (10 ng/ml) significantly enhanced the levels of 8-iso-PGF_2α_ (p < 0.001, n = 3) as compared with basal levels. Pre-incubation of cap reduced the levels of released 8-iso-PGF_2α_ in LPS activated microglia. This reduction in the levels was only achieved with the higher conc. of cap, 10 µM (mean 64.52 ± 9.90%, p < 0.05) and 25 µM (mean 52.11 ± 10.81%, p < 0.01). With the lower concentrations (0.1 & 1 µM) a tendency towards decrease was observed, but did not reach to statistical significant values as shown in Fig. 2b. To rule out whether these effects were due to the decrease in the viability of cells, ATP and lactate dehydrogenase (LDH) viability assays were performed. Obtained data revealed that all the concentrations (ranging from 0.01–25 µM, n = 3) had no adverse effects on ATP and released LDH levels, and thus on the viability of microglial cells. This suggests that the reduction in the levels of PGE_2_ and 8-iso-PGF_2α_ by cap was not due to cytotoxicity. To validate the functionality of this assay, higher conc. of DMSO (10%) was used which showed obvious cell death as depicted in Supplementary Fig. S2.

**Figure 3:**
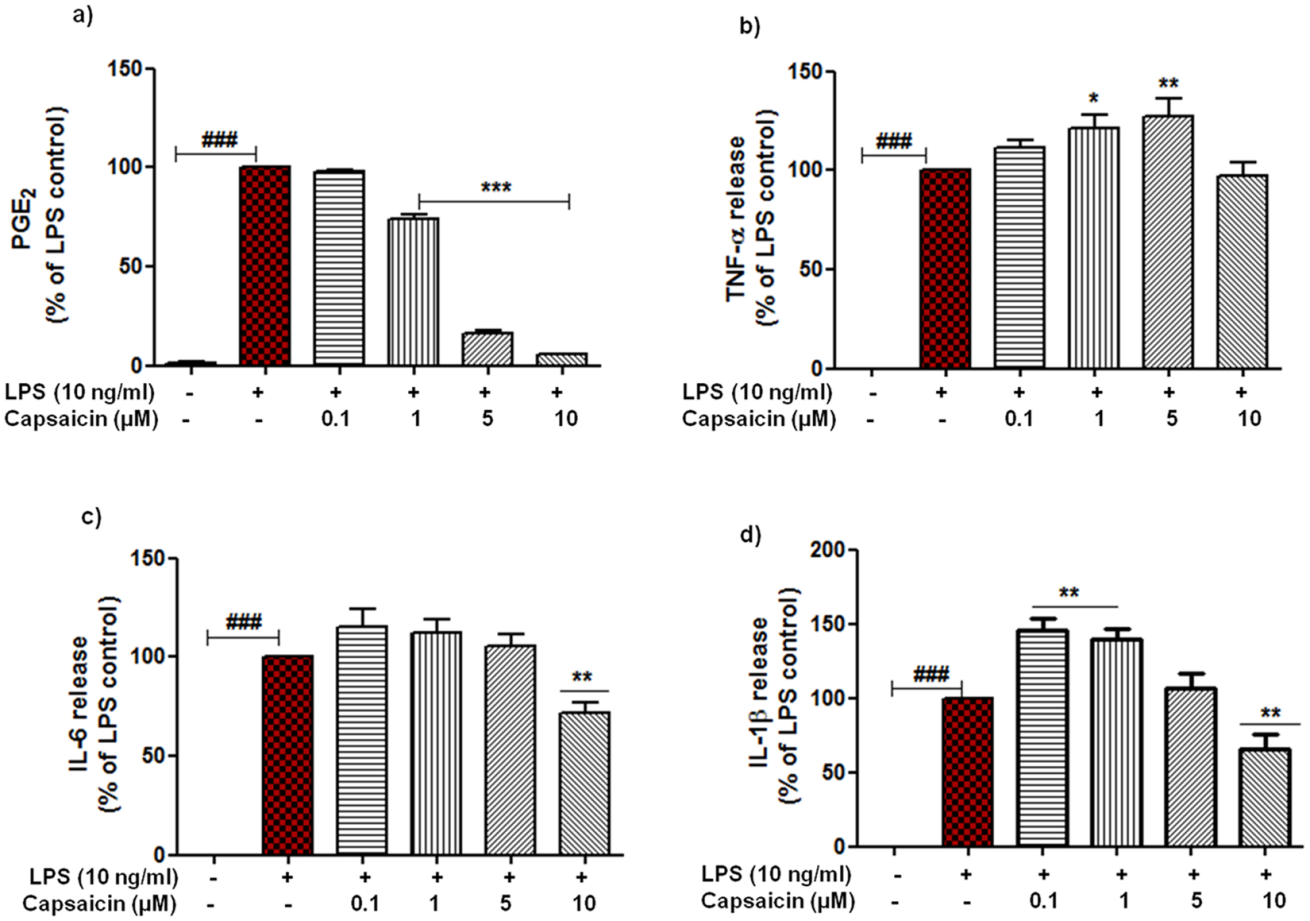
Regulation of inflammatory markers by capsaicin (cap) in activated primary human peripheral blood monocytes. Monocytes were pre-treated with cap (0.1–10 µM) for 30 min, afterwards cells were incubated with or without LPS (10 ng/ml) for the next 24 h. At the end of incubation, cell supernatants were collected, centrifuged and release of PGE_2_ (**a**), TNF-α (**b**), IL-6 (**c**) & IL-1β (**d**) were measured. Statistical analyses were carried out by using one way ANOVA with *post hoc* student Newman-Keuls test (Multiple comparisons). Results are expressed as means ± SE of 5 independent experiments. ^#^p < 0.05; ^##^p < 0.01; ^###^p < 0.001 compared with control cells. *p < 0.05; **p < 0.01; ***p < 0.001 compared with LPS (10 ng/ml) activated cells.

Of note, it has documented that blood monocytes infiltrate the CNS and differentiate into cells resembling phenotypically activated microglia. Thus, we envision that cap’s mode of action can be extrapolated in circulating monocytes. To this end, we studied the effects of cap on PGE_2_ and selected cytokines in activated primary human monocytes. LPS (10 ng/ml) substantially increased the production of PGE_2_ (p < 0.001, n = 5) as well as cytokines (p < 0.001, n = 5, Fig. 3). Intriguingly, pre-incubation of cap exhibited strong reduction in the release of PGE_2_ in LPS activated monocytes. Dampening in PGE_2_ release started with the dose of 1 µM (73.67 ± 2.90%, p < 0.001,) through 5 µM (16.33 ± 1.66%, p < 0.001) and reached maximal at 10 µM (5.66 ± 0.33%, p < 0.001) as shown in Fig. 3a. On the contrary, pre-incubation of cap exerted differential effects on the release of cytokines (3b–d). These data indicate that cap might have similar mode of action in the suppression of PGE_2_ in resident brain macrophages and in monocytes under inflammatory milieu.

### Pharmacological inhibition and genetic deficiency of TRPV1 did not counteract the suppression of PGE_2_ release by capsaicin in LPS-activated microglia

**Figure 4:**
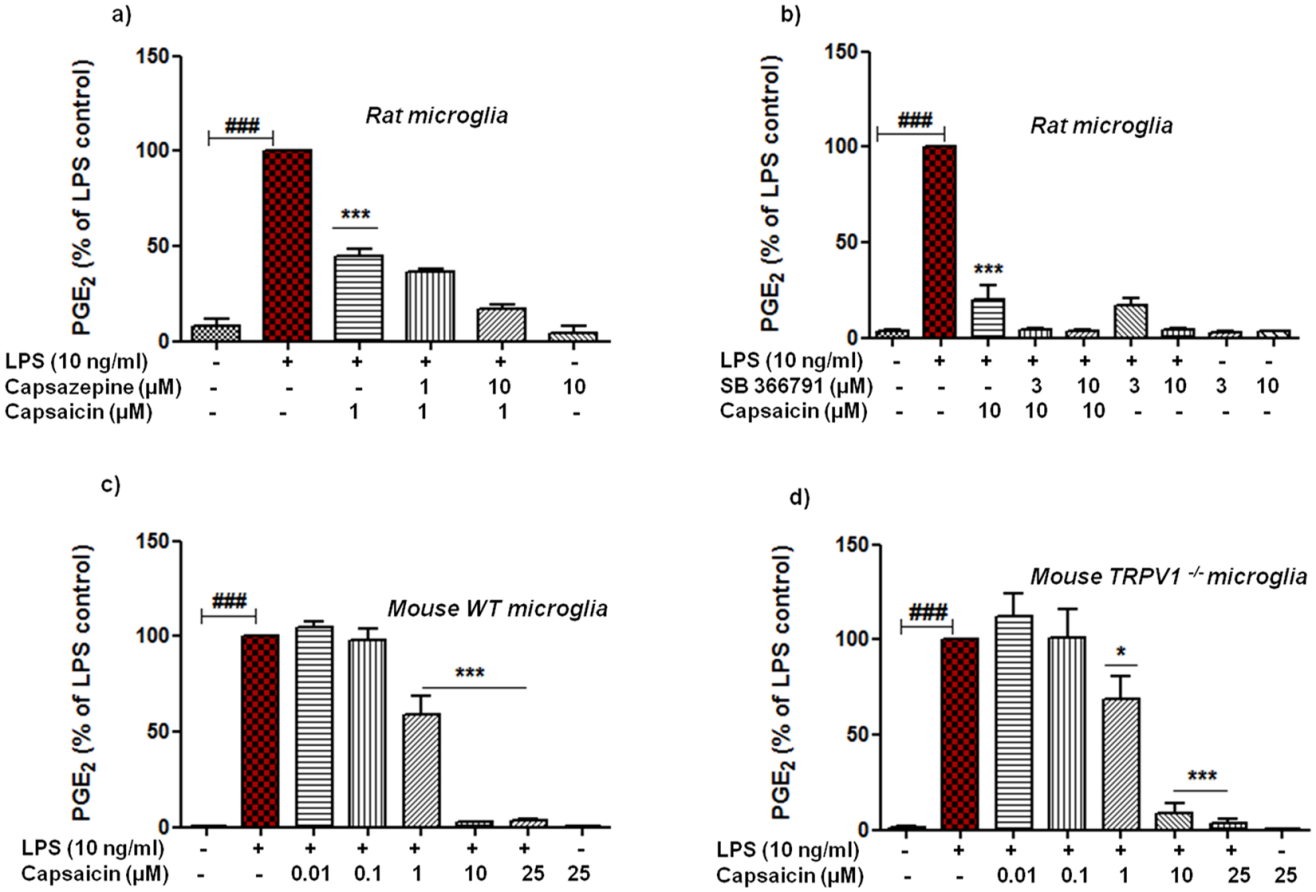
Effects of pharmacological inhibition and genetic deletion of TRPV1 in capsaicin (cap) treated activated microglia. Rat primary microglial cells were pre-treated either with capsazepine (**a**) or SB366791 (**b**) for 30 min thereafter cap was added for the next 30 min subsequently, cells were incubated with LPS (10 ng/ml) for 24 h. Mouse primary microglia were taken from either wild type (WT) animals (**c**) or from TRPV1^−/−^ animals (**d**). In both cases cells were pre-treated with cap (0.01–25 μM) for 30 min thereafter incubated with LPS (10 ng/ml) for the next 24 h. At the end of incubation, cell supernatants were collected and release of PGE_2_ was measured by EIA. Statistical analyses were carried out by using one way ANOVA with *post hoc* student Newman-Keuls test (Multiple comparisons). Results are expressed as means ± SE of three independent experiments. ^#^p < 0.05; ^##^p < 0.01; ^###^p < 0.001 compared with control cells. *p < 0.05; **p < 0.01; ***p < 0.001 compared with LPS (10 ng/ml) activated cells.

Firstly, we validated the expression of TRPV1 in microglia cells via qRT-PCR and immunoblotting as shown in Supplementary Data (Fig. S3). We detected the expression of TRPV1 in microglial cells, although the expression levels were weak. For the functionality of qPCR primers and western blot antibody, positive controls namely rat cortex and dorsal root ganglions (DRGs) were respectively, used. Secondly, we investigated whether putative receptor (TRPV1) of cap was involved in the release of PGE_2_. To this end, we used two well-accepted antagonists, capsazepine and SB366791. As a result, we did observe significant reduction (p < 0.001, n = 3–5) in the levels of PGE_2_ by cap in LPS activated rat microglia. Though these effects were not counteracted by prior application of any of the antagonists at given concentrations as shown in Fig. 4a and b. Rather, we observed that capsazepine as well as SB366791 itself inhibited the release of PGE_2_ in activated microglia, indicating a more complex antagonist-receptor interaction which was not further explored in our present study. However, these antagonists had no effect on the levels of PGE_2_ when used alone.

In order to confirm the data obtained from pharmacological inhibition, and rule out any desensitization of TRPV1 by prolonged exposure to cap, we performed similar experiments in microglia taken from global TRPV1 knock-out (TRPV1^−/−^) mice and compared the results with their wild type (wt) counterparts. Interestingly, we observed that cap again substantially (p < 0.001, n = 5) suppressed the release of PGE_2_ in activated microglia similar to wt mouse microglia (Fig. 4c and d). In case of cytokine release, differential regulation (a trend towards increase) was observed by cap, but did not reach to a statistical significant (p > 0.05, n = 4) value for TNF-α (Supplementary Fig. S4a,c). On the contrary, notable inhibition (p < 0.001, n = 4) of IL-6 (both in wt & TRPV^−/−^) was observed with highest conc. of cap, when compared with LPS, as is shown in Supplementary Fig. S4b,d. These data suggests that cap may regulate the release of PGE_2_ and cytokines in activated microglia via TRPV1 independent mechanisms.

### Capsaicin significantly reduced the LPS-induced PGE_2_ release in organotypic hippocampal slice cultures (OHSCs)

**Figure 5:**
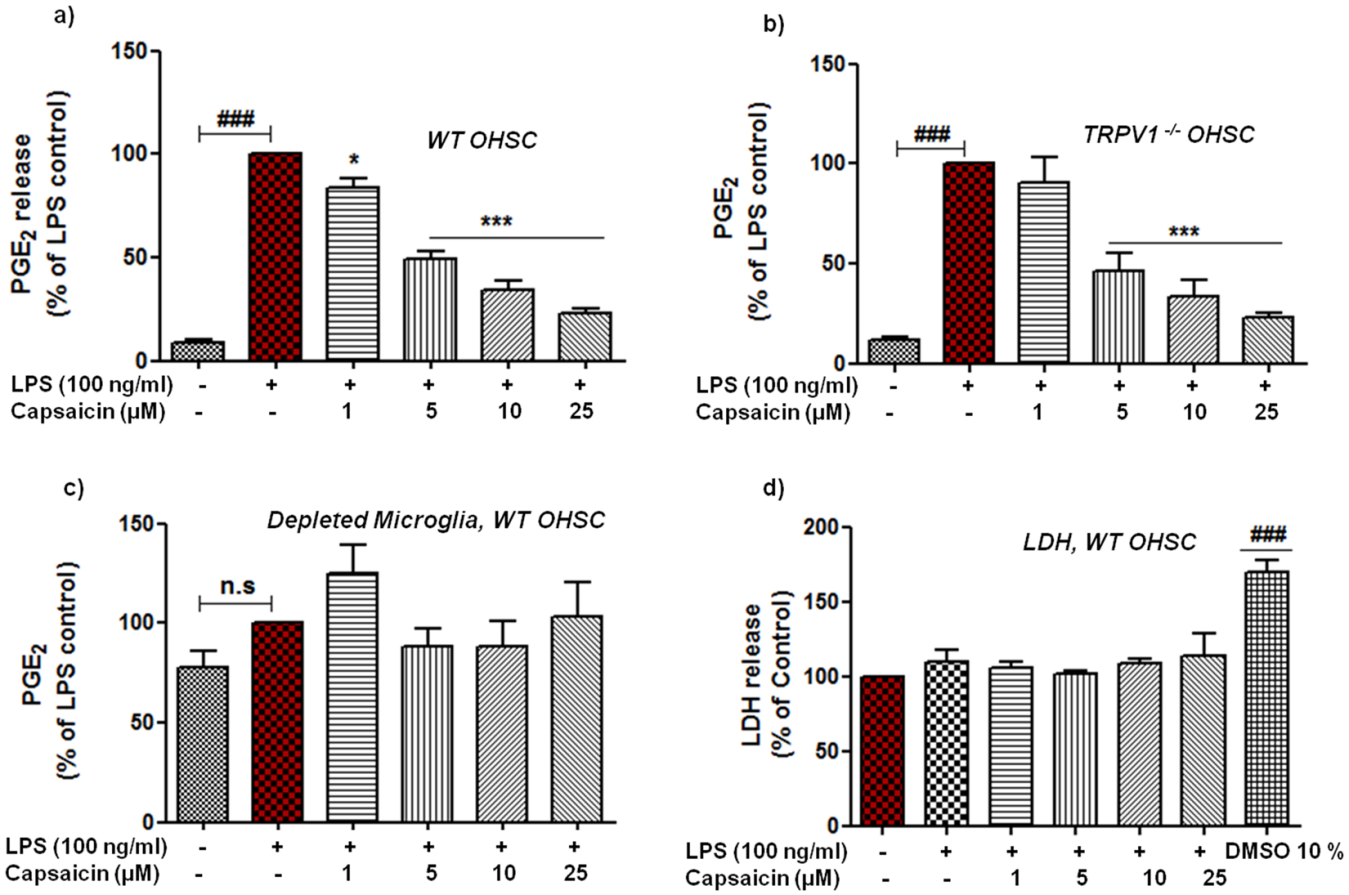
Capsaicin (cap) inhibits the release of PGE_2_ in LPS activated OHSCs without affecting the viability of cells. Slices were pre-treated with cap (1–25 µM) for 30 min followed by incubation with or without LPS (100 ng/ml) for the next 24 h. Mouse slices were either taken from wild type (wt) animals (**a**) or from TRPV1^−/−^ animals (**b**). Influence of cap (1–25 µM) on the release of PGE_2_ in microglia-depleted slices (**c**). Cap did not exert any adverse effects on viability of cells in LPS-stimulated slice cultures (**d**). Lactate dehydrogenase (LDH) assay was performed in tissue supernatants to assess the cellular viability after cap (1–25 µM) treatment in the absence or presence of LPS. Statistical analyses were carried out by using one way ANOVA with *post hoc* student Newman-Keuls test (Multiple comparisons). Results are expressed as means ± SE of three independent experiments. ^###^p < 0.001 compared with control cells. *p < 0.05; **p < 0.01; **p < 0.001 compared with LPS (100 ng/ml) activated cells.

Next, we examined the potential interference of cap in more robust multicellular model of hippocampal slice cultures. OHSCs serve as *in vitro* explant culture that reflects many aspects of the hippocampus *in vivo* situation by maintaining a certain degree of intrinsic connectivity and lamination^45^. Moreover, OHSCs retain the complex three-dimensional (3D) organization of the hippocampus and being utilized to study and understand various aspects of CNS associated pathologies^46, 47^. Of note, this model has been successfully utilized for studying microglial polarization in the presence of surrounding neuronal and glial cells^48^. Morphologically intact hippocampi can be immunostained and visualized for neuronal and microglial population (Supplementary Fig. S5). To this end, we investigated the possible regulatory effects of cap in slices prepared from wt and TRPV1^−/−^ mice. After 24 h incubation of slices with LPS (100 ng/ml), a significant (p < 0.001, n = 4) release of PGE_2_ (Fig. 5a), TNF-α and IL-6 (p < 0.001, n = 3, Supplementary Fig. S6a,b) was observed in wt microglia, validating the functionality of this model in context of neuroinflammation. Interestingly, prior incubation of OHSCs with cap successfully suppressed the LPS-activated release of PGE_2_ without affecting the viability of tissue cultures measured as LDH release in slice supernatants (Fig. 5d). The reduction in the release of PGE_2_ started at 1 μM (mean 84.03 ± 4.20%, p < 0.05, n = 4), followed by 5 μM (mean 49.51 ± 3.62%, p < 0.001), 10 μM (mean 34.59 ± 4.47%, p < 0.001) and 25 μM (mean 22.78 ± 2.11%, p < 0.001). On the contrary, cap failed to alter cytokine production, with only a marginal enhancement observed in the release of TNF-α and IL-6 (Supplementary Fig. 6), similar to mouse microglial monocultures. Thereafter, experiments utilized slices taken from global TRPV1^−/−^ mice to pinpoint the possible function of this receptor in neuroinflammation. Similarly, we did observe significant inhibition (p < 0.001, n = 4) in the release of PGE_2_ (Fig. 5b). Again, absence of TRPV1 did not influence the potent inhibitory action of cap on the release of PGE_2_ in this multicellular model of inflammation, confirming our data obtained from microglial monocultures. Additionally, we performed experiments in OHSCs depleted microglia to observe the relevance and contribution of microglia in context of action of cap in neuroinflammation^49^. To this end, we observed diminished induction of PGE_2_ (mean control 77.75 ± 7.88%, p > 0.05, n = 3) & cytokines when stimulated with LPS (100%). Moreover, cap did not significantly affect the levels of PGE_2_ in LPS activated slices (Fig. 5c), indicating that microglia are the main contributor to the effects of cap in this model of neuroinflammation.

### Capsaicin suppresses the PGE_2_ production by inhibiting the COX-2 and mPGES-1 gene expression and protein levels in LPS-activated microglia

**Figure 6:**
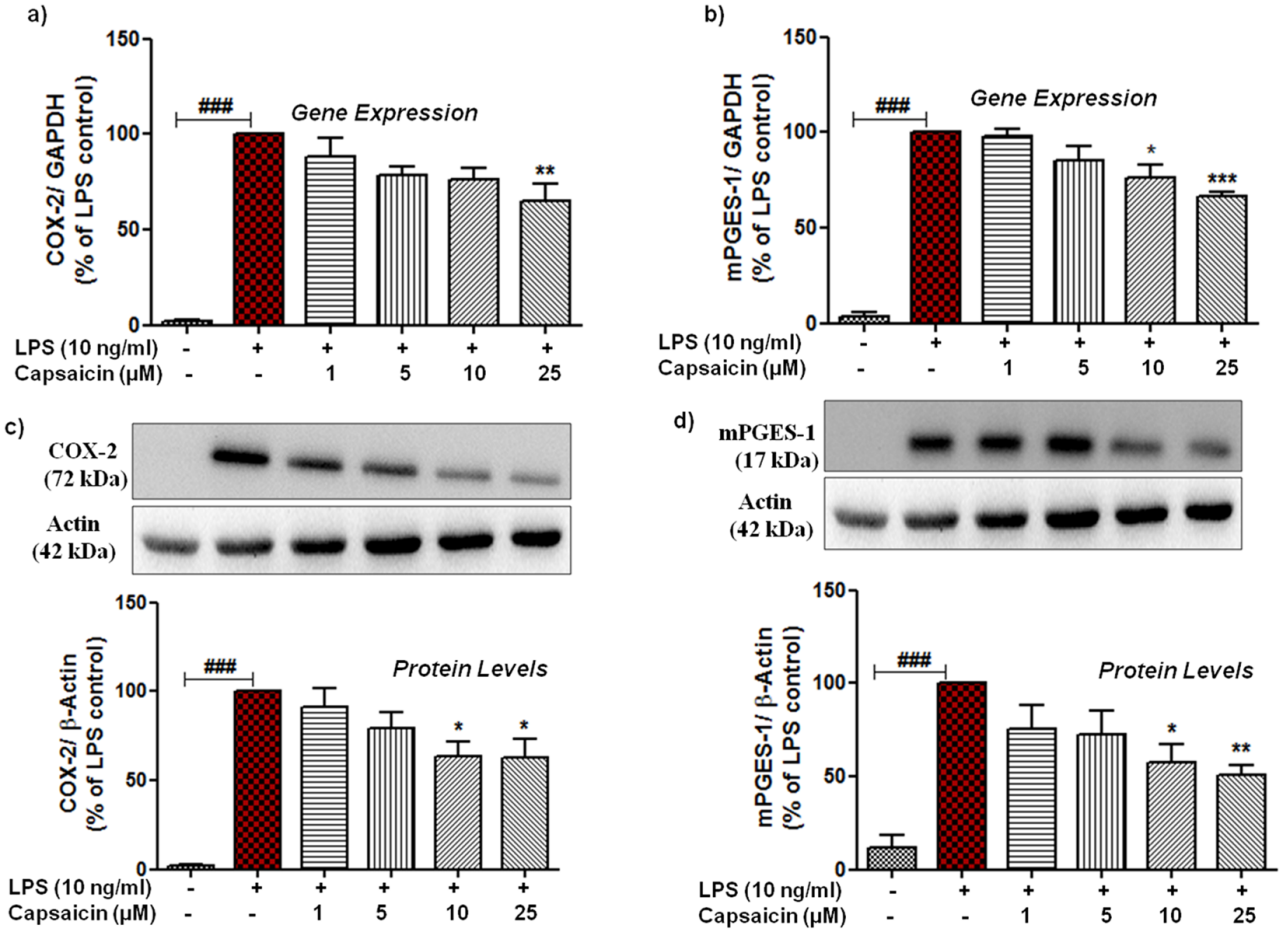
Effects of capsaicin (cap) on gene expression of COX-2 and mPGES-1 in LPS-activated microglia. Effects of cap on protein level of COX-2 and mPGES-1. Cells were pre-treated with cap (1–25 µM) for 30 min followed by incubation of cells with or without LPS (10 ng/ml) for the next 4 h. Gene expression of COX-2 and mPGES-1 was studied. Afterwards, gene expression of (**a**) COX-2 and (**b**) mPGES-1, was analysed by real-time quantitative PCR. GAPDH was used as an internal control for normalisation and data were quantified by using comparative cycle threshold Ct method. Similarly, cells were treated with cap (1–25 µM) for 30 min thereafter incubated with or without LPS (10 ng/ml) for 24 h. Whole cell lysates were subjected to western blot for COX-2, mPGES-1 and Actin. After the successful transfer of protein, membrane was blocked and eventually cropped horizontally into three pieces according to the target protein. First between 55 and 100 kda (for COX-2, 72 kda), second between 35 and 55 kda (for Actin, 42 kda) and third between 10 and 25 kda (for mPGES-1, 17 kda). Representative blots for COX-2, mPGES-1 and Actin are shown (c & d upper panel) and densitometry analyses were performed (**c** and **d**, lower panel). Representative original blots are included in Supplementary Information (Fig. S10). To confirm equal sample loading, β-actin were used for normalisation. Moreover, data are presented as percentage control of LPS. Statistical analyses were carried out by using one way ANOVA with *post hoc* student Newman-Keuls test (multiple comparisons). Results are expressed as means ± SE of 3–5 independent experiments. *p < 0.05; **p < 0.01; ***p < 0.001 compared with LPS (10 ng/ml) activated cells. ^#^p < 0.05; ^##^p < 0.01; ^###^p < 0.001 compared with control cells.

PGE_2_ synthesis occurs during neuroinflammation through the enzymatic action of COX-2 and mPGES-1. mPGES-1 is known to be coupled to COX-2 in most of the models in the biosynthesis of PGE_2_. Consequently, we determined whether the effect of cap on PGE_2_ is mediated through direct inhibition of the synthesis of these enzymes. Addition of LPS to microglia cultures strongly induced gene (Fig. 6a,b) expression as well as protein (Fig. 6c,d) levels of COX-2 and mPGES-1 as compared with controls. Reduction in the gene expression and protein levels of COX-2 by cap was observed, ranging from concentrations of 1–25 μM, but a statistically significant value was only achieved at the higher conc. For COX-2 gene expression, significant inhibition was at 25 μM (mean 64.70 ± 9.58%, p < 0.01, n = 6) although for protein levels, cap conferred its effects at 10 μM (mean 63.55 ± 9.19%, p < 0.05, n = 7) and 25 μM (mean 62.61 ± 10.69%, p < 0.05). On the contrary, significant inhibition of mPGES-1 gene expression was started already with the conc. of 10 µM (mean 69.07 ± 9.28%, p < 0.05, n = 4) and a maximal effect was observed at 25 µM (mean 23.99 ± 4.40%, p < 0.001) as shown in Fig. 6b. Similar to COX-2 protein data, reduction in mPGES-1 immunoreactivity was also observed at 10 µM (mean 57.25 ± 9.94%, p < 0.05, n = 6) and 25 µM (mean 51.02 ± 4.67%, p < 0.01). These data suggest that the reduction in the release of PGE_2_ was dependent, at least in parts, on the inhibition of COX-2 and mPGES-1. However, data on COX-2 and mPGES-1 alone could not justify the robust effects of cap on the PGE_2_ release.

### Effects of capsaicin on PGE_2_ were independent of cytosolic phospholipase A2 (cPLA2), COX-1 protein levels and COX enzymatic activity

Previously, we showed that the cPLA2 inhibitor strongly reduced the LPS-induced PGE_2_ release in rat microglia without affecting the COX-1/2 synthesis^18^. Therefore, we wonder whether cap exerted its robust effects on PGE_2_ via directly affecting the activation and synthesis of cPLA2. To this end, we observed a remarkable increase in the activation of cPLA2 as depicted in Supplementary Data (Fig. S7a, upper band) when stimulated with LPS (p < 0.05, n = 4), leaving synthesis of total cPLA2 unchanged (lower band). Pre-treatment of cap (1–25 µM) was not able to affect either the activation, or the total synthesis, of cPLA2 in LPS activated microglia, suggesting that the reduction of PGE_2_ by cap was not by interfering in the release of AA from the plasma membrane of microglia. Furthermore, we also investigated whether cap affected the COX-1 synthesis as the expression and function of this enzyme is also been debated and studied in inflammatory related disease conditions^50, 51^. Neither LPS nor the cap pretreated microglial cells showed any change (p > 0.05, n = 4) in the protein levels of COX-1 (Supplement Fig. S7b), validating our previous study^44^.

We observed robust effects of cap on the PGE_2_ release ranging from nanomolar concentrations but the effects on the COX-2/mPGES-1 immunoreactivity were not that potent. Thus, we extended our study to investigate if these robust inhibitory effects of cap on PGE_2_ release were due to a direct suppression of COX enzymatic activity. To this end, we studied the effects of cap on both COX-1 and COX (COX-1 + COX-2) enzymatic activity in microglia. Supplementary Figure S8a and b shows the findings from both enzymatic activity assays. We observed that AA remarkably increased (p < 0.001, n = 3) the release of PGE_2_, hence COX activity, in the LPS (10 ng/ml) treated microglia (taken as 100%) when compared with control cells or with AA (15 μM) or LPS (10 ng/ml) alone. However, cap failed to exert any significant change in the COX activity. None of the used conc. affected the released amount of PGE_2_ substantially, except a tendency towards reduction was observed with the higher concentrations. We observed that diclofenac (10 μM), a known COX inhibitor could significantly (p < 0.001, n = 3) inhibit the COX mediated PGE_2_, validating the functionality of our assay (Fig. S8a). Furthermore, in case of COX-1 activity, AA substrate significantly (p < 0.001, n = 3) induced the enzymatic activity, but cap did not influence the release of COX-1 mediated PGE_2_. Application of specific COX-1 inhibitor (SC 560 1 μM) did strongly suppress (p < 0.001, n = 3) the production of PGE_2_, confirming the validity of this system to study the potential inhibitory properties of cap on microglial COX enzymatic activity (Supplement Fig. S8b). This data set ruled out the involvement of possible direct effects of cap on COX enzymatic activities.

### Anti-inflammatory action of capsaicin is mediated by inhibition of p38 activation and its downstream kinase MK2 but not NF-κB in the microglia

**Figure 7:**
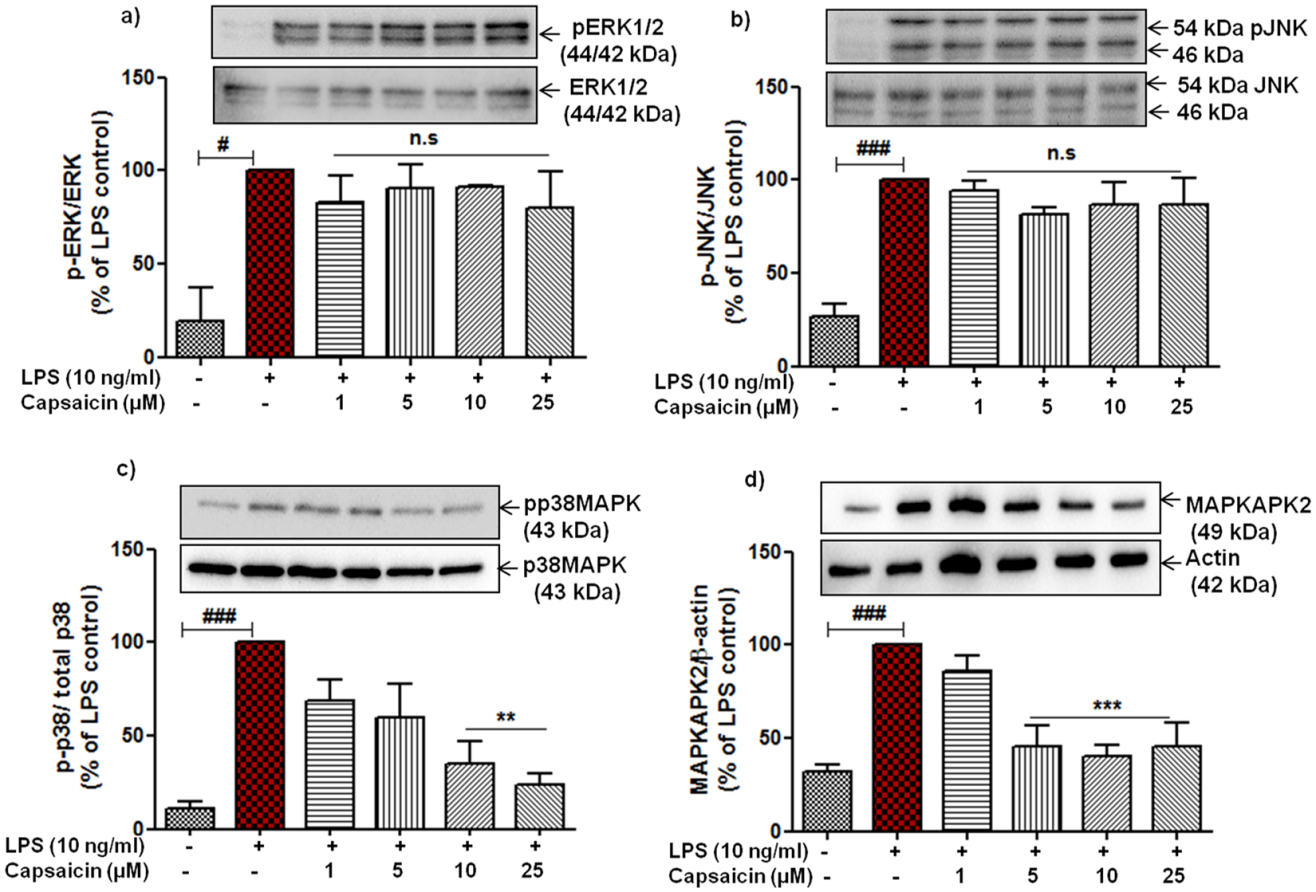
Effects of capsaicin (cap) on the activation of ERK 1/2, JNK, p38 and MK2. Cells were pre-treated with cap (1–25 µM) for 30 min followed by stimulation with or without LPS (10 ng/ml) for 30 min. Whole cell lysates were subjected to western blots analyses. After the successful transfer of protein, membranes were blocked and eventually cropped horizontally between 60 kda and 35 kda (depending on the size of protein) to get all the target proteins. Representative blots (upper panel) and densitometry analyses (lower panel) are shown (**a**) ERK1/2, (**b**) JNK, (**c**) p38 MAPK, and (**d**) MAPKAPK2 or MK2. Representative original blots are included in Supplementary Fig. S10. Statistical analyses were carried out by using one way ANOVA with *post hoc* student Newman-Keuls test (multiple comparisons). Results are expressed as means ± SE of 3–4 independent experiments. ^#^p < 0.05; ^###^p < 0.001 compared with control cells. **p < 0.01, ***p < 0.01 compared with LPS (10 ng/ml) activated cells.

The role of mitogen-activated protein kinases (MAPKs) in neuroinflammation and neurodegenerative diseases is well established^23^. The major MAPK pathway subfamilies involved in the regulation of COX-2/mPGES-1 and cytokine synthesis in LPS activated microglia are signaling proteins such as p38 MAPK, extracellular signal regulated kinases (ERK1/2) or (p44/42 MAPK), and c-jun-N terminal kinase (JNK). Therefore, we studied the possible effects of cap on the phosphorylation of ERK1/2, JNK, p38 MAPK and its downstream kinase MAPKAPK2 (MK2) in primary microglia. Stimulation with LPS for 30 min significantly phosphorylated, and thus activated, ERK1/2, JNK, p38 MAPK and MK2 (Fig. 7). Pre-incubation of cap with various concentrations (1–25 µM) for 30 min did not alter LPS activated ERK and JNK as shown in Fig. 7a and b. Interestingly, treatment of cap at the concentrations of 10 μM (mean 35.03 ± 11.78%, p < 0.01, n = 3) and 25 μM (mean 23.57 ± 5.91%, p < 0.01), showed significant reduction in the phosphorylation of p38 MAPK without affecting the synthesis of total p38 MAPK in activated microglia (Fig. 7c). A trend towards a reduction in phosphorylation with lower concentrations (at 1 µM, mean 69.11 ± 10.79%, p > 0.05; at 5 µM, mean 59.98 ± 17.83%, p > 0.05) was already observed but did not reach to a statistical significant value. Moreover, we also investigated the potential effects of cap on the downstream kinase of p38 namely MK2. Findings in Fig. 7d depict that prior incubation of cap for 30 min strongly reduce the LPS-induced activation of MK2 in microglia. Strong inhibitory effects start with the dose of 5 μM (mean 45.84 ± 10.71%, p < 0.001, n = 4), and reached maximal at 10 µM (mean 40.55 ± 6.01%, p < 0.001), indicating the specificity of cap for MK2 at much lower concentrations when compared with p38 MAPK inactivation.

One of the first responses of the microglia to LPS stimulation is the phosphorylation, and degradation, of IκB. The decay of IκB-α is used as an indicator of NF-κB activation^52^. Therefore, we also studied the effects of cap on the degradation of IκB-α. Stimulation of LPS (10 ng/ml) for 15 min led to the degradation of IκB-α, while pre-incubation with cap for 30 min failed to revert this effect (Supplement Fig. S9), suggesting that the anti-neuroinflammatory effects exhibited by cap are confined to the MAPK signaling pathway and are independent of the NF- κB pathway.

## Discussion

In the present study, we demonstrate the novel anti-inflammatory effects of cap in activated microglia. In particular, cap strongly suppressed the production of PGE_2_ and free radical (8-iso-PGF2α) formation. Pharmacological blockade and genetic depletion of TRPV1 showed that the effects of cap were independent of its putative receptor, but dependent on the direct inactivation of the p38MAPK-MK2-COX-2/mPGES-1 pathway. Moreover, suppression of PGE_2_ release was confirmed in multicellular models of hippocampal slice cultures, as well as in human primary blood monocytes. To the best of our knowledge, this is the very first evidence for the TRPV1-independent anti-inflammatory effects of cap in the resident immune cells of CNS. To date, it has been well accepted that TRPV1 is the putative receptor of cap. Above all, expression and function of TRPV1 in the brain is still under debate. Earlier, a TRPV1-specific antibody and RT-PCR investigation confirmed the expression of TRPV1 in the hippocampus, hypothalamus and cortex^53, 54^. In contrast, recent use of TRPV1 reporter mice has revolutionized the study of TRPV1 expression by suggesting that the expression of this receptor is minimal within a few discrete brain regions^55^. Moreover, other than plasma membrane, presence of functional TRPV1 in intracellular organelles has also been documented^38^. In our study, presence of weak microglial TRPV1 has been observed by using qRT-PCR and specific antibody detection method (Supplementary Fig. S3), which confirms previous studies^38, 40^. Few lines of evidence has demonstrated that the activation of TRPV1-stimulates Ca^2+^ influx induced cellular death in variety of cell types, including microglia and neurons^40, 56^. We observed that cap (0.01–25 µM) did not exert any adverse effects on the viability of activated microglial cells and OHSCs, indicating that the observed inhibition of PGE_2_ and 8-iso-PGF2_α_ were independent of potential toxicity of cap. These differences between our study and above-mentioned study by Kim SR *et al*. might be due to the use of lower concentrations (0.01–25 µM) vs very high concentrations (50–100 µM) of cap respectively. Additionally, authors showed that 10 µM of cap (conc. where we observed most of the effects) did not affect the viability of neurons as well as microglia, further confirming our data. Next, we found that the inhibitory effects of cap on the release of PGE_2_ were dependent, at least in parts, on preventing the synthesis of COX-2/mPGES-1 enzymes. Overexpression of COX-2 and/or mPGES-1 has been linked to many brain pathologies^57–59^. Thus, novel compounds with the potency to interfere with this pathway warrant investigation. Our previous work has shown that natural compounds with anti-oxidant activity have the ability to regulate the microglial activation via interfering with COX-2/mPGES-1 synthesis^21, 44^. In agreement to this, the data presented here also showed the inhibitory effects of cap on COX-2 and mPGES-1, though the inhibition was not as robust as observed in inhibition of PGE_2_. Of high interest, cap showed rather stronger inhibition on mPGES-1 gene expression and protein levels, pointing towards the beneficial feature of this compound when compared with existing non-selective COX inhibitors. Immunomodulatory effects of cap in the peripheral nervous system (PNS) have previously been explored^60, 61^. However, most of these studies were limited to murine peripheral macrophages where authors showed the anti-inflammatory effects by targeting COX-2/PGE_2_ synthesis without studying terminal enzyme (mPGES-1) in PGE_2_ synthesis. In an another study, effects of TRPV1 agonists, other than cap, have also been explored in microglia- like cells^62^. In accordance with our current study, all of these studies reported that the mode of action of cap was not dependent on the TRPV1 receptor. Next, we extended our study to look if the robust inhibitory effects of cap on PGE_2_ release can be explained via studying either cPLA2 synthesis/activation or COX enzymatic activity. In the biosynthetic pathway leading to PGE_2_, arachidonic acid (AA) liberated from the membrane phospholipids, by the action of phospholipases A2 (PLA_2_), converts to PGH_2_ by COX-1 or COX-2 and then isomerised to PGE_2_ by terminal prostaglandin E synthases (PGES). Earlier we showed that LPS is capable of inducing the activation of cPLA_2_. Moreover, a cPLA2 inhibitor strongly reduced the LPS-induced PGE_2_ release in rat microglia, without affecting the COX-1/2 synthesis^18^. Therefore, we considered whether cap exerted its robust effects on PGE_2_ via directly affecting the activation of cPLA2. To this end, we studied both the activation and the total synthesis of cPLA_2_. Blot analysis (Supplementary Fig. S7a) clearly indicates that the LPS up regulated the activity of cPLA2 without affecting the total synthesis of this enzyme. However, cap failed to alter LPS induced activation/synthesis of cPLA2, indicating that it does not interfere in the release mechanisms of AA from phospholipids. Another possibility is that cap acts on the catalytic site of COX-2, which is the primary mechanism of action of drugs like aspirin, as discussed in our earlier study^63^. Nevertheless, we observed only a tendency towards an inhibition in total COX activity with higher concentrations of cap (10 & 25 µM) which did not reach to statistically significant values. In the case of COX-1 activity, we observed a trend towards reduction with the highest conc. (25 µM), as depicted in the Supplementary Fig. S8a,b. Thus, these data ruled out the possible direct effects of cap (at least at lower concentrations) on COX enzymatic activities.

Previously, we have documented that MAP kinases and NF-κB play crucial roles in regulating the transcriptional activity of microglial genes related to neuroinflammatory events^64^. Of significance is the regulatory role of p38 MAPK, and its downstream substrate MAPKAPK2 or MK2, in chronic inflammatory associated diseases such as cancer, pulmonary diseases, metabolic dysfunction, chronic pain, cerebral ischemia, multiple sclerosis (MS), Alzheimer’s and age-related disorders^22, 24, 65, 66^. Consequently, we investigated whether inhibition of p38 MAPK, NF-κB and other kinase activities contributed to the anti-inflammatory effects of cap. We did observe marked reduction in the phosphorylation, and hence activation, of p38 MAPK by cap in activated microglia without altering the IκB-α and other kinases (pERK & pJNK). Encouraged by the observation that cap could block the activation of p38 MAPK, we went further to investigate whether cap prevents the activation of MK2, a downstream kinase of p38 MAPK. Indeed, we found that cap exhibited significant changes in the activation of MK2 in LPS-activated microglia. More interestingly, cap suppressed the activity of MK2 at lower concentrations, indicating a more pronounced effect than on the p38 MAPK. All of these observations suggest that cap produces anti-neuroinflammatory action in microglia by targeting the p38 MAPK-MK2-PGE_2_ axis.

Under homeostatic conditions, microglia express surface markers typically present on many other tissue macrophages and/or monocytes, including CD11b, F4/80, CD115 and ionized calcium-binding adapter molecule (Iba-I)^67^. Although microglial homeostasis is maintained through local self-renewal, and microglia are ontogenically distinct^68^ from other myeloid cells, circulating precursors can give rise to microglia-like cells under certain circumstances^69^. In this study, the author identified a specific monocyte subpopulation, namely Ly-6C^hi^Gr-1^+^CCR2^+^CX3CR1^lo^ cells, as the precursor of adult murine microglia in the peripheral blood. Ly-6C^hi^ CCR2^+^ monocytes specifically accumulated in CNS lesions and differentiated into brain-specific macrophages, the microglia. Keeping this in mind, we extended our findings to extrapolate the effects of cap in primary human blood monocytes. To this end, we studied the possible effects of cap mainly on PGE_2_ and cytokine production. Of high relevance, we observed that cap produced a substantial reduction in the release of PGE_2_ in LPS activated monocytes, further confirming our data obtained from resident microglia and OHSCs. On the contrary, levels of cytokines (TNF-α & IL-1β) were further increased with lower doses of cap and substantially decreased (IL-6 & IL-1β) with the highest dose. These data were in some aspects similar to our above findings in mouse mono and multicutures of microglia, but not identical. Most recently, peripheral blood mononuclear cells and U-937 macrophages were utilized for studying the effects of cap, and its analogue nonivamide, on cytokine synthesis^70^. In contrast, authors showed the inhibitory action of both compounds in LPS induced IL-6 and TNF-α in PBMCs as well as U-937 macrophages, suggesting that these effects might be cell specific (PBMC or macrophages vs isolated monocytes). Moreover, we consider that regulation of cytokine synthesis by cap might target different signaling pathways, and warrants further investigations in the context of neuroinflammation. Additionally, it remains an obscurity in our current study whether cap interfered in the p38 MAPK/MK2/PGE_2_ axis via other receptors, either related to the TRP family members, or other microglial channels including potassium (K^+^)^71, 72^. For instance, one very recent finding showed that KV 1.3 and KCa 3.1 blockers inhibited pro-inflammatory cytokine production, iNOS and COX-2 expression, demonstrating the indispensable role of these channels in microglia activation^73, 74^. Furthermore, agonists of TRPV members have proven to be modulators of K^+^ currents, and thereby regulate microglia activation^75^. Thus, one cannot rule out the possibility of involvement of channels, other than TRPV1, in the action of cap. That is the very reason that in our ongoing studies, we are trying to address some of these unresolved questions.

Altogether, our current study presents the novel molecular targets of capsaicin, independent of TRPV1, in the synthesis and release of pro-inflammatory mediators within activated microglia. These findings have broad relevance in understanding, and paving new avenues for ongoing TRPV1 based drug therapies in CNS and periphery, in the context of dysregulated inflammatory-associated diseases. To date, most of the clinical applications of capsaicin are restricted to pain management. Further investigations are required to reveal pharmacological features of capsaicin in other pathological conditions. Moreover, our current study provides impetus to investigate current TRPV1 based agonist/antagonist in context of neuroinflammation in various cellular and animal models. It is very well possible that these molecules might modulate exaggerated inflammatory events by targeting either directly or via other ion channels, other than the TRP family members.

## Materials and Methods

### Animal and human study

The ethics committee of University of Freiburg Medical School, Freiburg, Germany, approved all experimental protocols (both animal and human). All experimental procedures were conducted according to the guidelines and regulations of ethic committee of University of Freiburg Medical School. Maximum efforts were made to minimize the number of animals used, and their suffering during this study.

Animals were housed in a vivarium with a 12-h light–dark cycle, food and water *ad libitum*, and were obtained from the Center for experimental models and transgenic services-Freiburg (CEMT-FR). C57BL/6 mice served as wild-type controls, and were purchased regularly from Charles River to avoid genetic drift due to inbreeding. TRPV1^−/−^ mice (Strain *B6*.*129* × *1*-*trpv1tm1Jul*/*J*) were purchased from Jackson Laboratory (stock number 003770).

### Chemicals

Capsaicin (cap) and lipopolysaccharide (LPS, *Salmonella typhimurium*) (Sigma Aldrich, Deissenhofen, Germany) were dissolved in ethanol and phosphate buffered saline (PBS) as 100 mM and 5 mg/ml stock respectively. Clodronate disodium salt (Merck Chemicals GmbH, Darmstadt, Germany) was dissolved in ultra pure water (Biochrom, Berlin, Germany) as stock solution of 1 mg/ml. Capsazepine and SB366791 (Tocris, Wiesbaden-Nordenstadt, Germany) were dissolved in DMSO as 100 mM stock solutions.

### Primary microglia culture preparation from rodents

Primary mixed glial cell cultures were established from cerebral cortices of 1 to 3-day-old neonatal Sprague–Dawley rats, C57BL/6 wild type and TRPV1^−/−^ mice as described in our previous studies^52^. In brief, brains were carefully extracted, cerebral cortices were collected and freed from meninges. Forebrains were then minced and gently dissociated by repeated pipetting in Dulbecco’s modified eagle’s medium (DMEM) and filtered by passing through 70 µm nylon cell strainer (BD biosciences, Heidelberg, Germany). Cells were collected by centrifugation (1000 g, 10 min) and resuspended in DMEM containing 10% fetal calf serum (FCS) (GE Healthcare, Germany) and antibiotics 1% penicillin and streptomycin (40 U/ml and 40 µg/ml, respectively) (Sigma Aldrich, Germany). Cells were then cultured on 10 cm cell culture dishes (Falcon, Heidelberg, Germany) with the density of 5 × 10^5^ cells/ml in 5% CO_2_ at 37 °C. After 12–14 days *in vitro*, floating microglia were harvested from mixed glia (astrocyte-microglia) cultures, and then re-seeded into cell culture plates at the density of 2 × 10^5^ cells/well. On the next day, medium was discarded to remove non-adherent cells and fresh medium added, and after 1 h cells were used for experiments.

### Organotypic hippocampal slice cultures (OHSCs) preparation

OHSCs were prepared from mice as previously described^64^. Briefly, 1 to 3-day-old C57BL/6 mice pups were killed by decapitation and the hippocampi were dissected out and placed in ice cold Hank’s balanced salt solution (HBSS) supplemented with 15 mM HEPES and 0.5% glucose. Using a tissue chopper (McIlwain™), 375-μm-thick hippocampal slices were cut transversely. Slices (n = 5–6 hippocampi/well) with complete dentate gyri and CA3 and CA1 areas were placed onto semiporous Millicell-CM inserts (0.4 µm pore size; Millipore) and cultured according to the interface method^76^ in 6-well plates containing 1.2 mL culture medium (0.5 × MEM, 25% BME, 25% horse serum, 2 mM glutamax, 0.65% glucose; pH 7.2) at 35 °C, humidified atmosphere, 5% CO_2_. Medium changed every other day, and experiments were performed after 6 days *in vitro* (DIV).

To deplete microglia from OHSCs, the slice cultures were placed immediately after preparation on culture medium containing 100 µg/mL clodronate disodium salt^49^. After 24 h of preparation, the cultures were briefly washed with pre-warmed PBS and the cell culture inserts were placed on fresh culture medium. Similarly, medium was changed every other day, and experiments were performed after 6 days DIV.

### Isolation of human peripheral monocytes

Blood (buffy coat) from five healthy human donors was obtained from blood donation center at the University Medical Center, Freiburg after prior approval and informed consent. All methods were performed in accordance with the guidelines and regulations of ethic committee of University of Freiburg Medical School. For the extraction of monocytes, a standardized protocol (Ficoll gradient preparation, GE Healthcare, Freiburg, Germany) using a completely endotoxin-free cultivation was used, as previously described^77^. Briefly, using 50 ml tubes, 25 ml Ficoll was loaded with 25 ml blood (buffy coats) from healthy blood donors. The gradient was then established by centrifugation at 1800 rpm and 20 °C for 40 min with slow acceleration and deceleration. Peripheral blood mononuclear cells in the interphase were carefully removed and resuspended in 50 ml pre-warmed PBS (Cell Concepts, Umkirch, Germany), followed by centrifugation for 10 min at 1600 rpm and 20 °C. The supernatant was discarded and the pellet was washed in 50 ml PBS and centrifuged as described above. The pellet was then resuspended in 50 ml RPMI-1640 medium supplemented with 10% human serum (PAA Coelbe, Germany). After counting the number of cells in a particle counter (Euro Diagnostics, Krefeld, Germany), cells were seeded in 24-well plates for ELISA (1.5–2.0 × 10^5^ cells/well) and incubated at 37 °C, 5% CO_2_ for 1 h. Thereafter, the medium and the non-adherent cells (lymphocytes) were removed and fresh RPMI-1640 medium containing 1% human serum was added. Enriched monocytes were thus ready to be used for the experiments.

### Determination of PGE_2_ and 8-iso-PGF_2α_ production

Cultured primary microglia, OHSCs and human monocytes were pre-incubated with various conc. of cap for 30 min, thereafter cells were treated with or without LPS at indicated concentrations for the next 24 h. After the end of the incubation period, supernatants were collected, and then centrifuged at 1000 g for 5 min at 4 °C. PGE_2_ and 8-iso-PGF_2α_ production were assessed in the supernatants with enzyme immunoassay (EIA) kits purchased from Biotrend, Cologne, Germany and Cayman Chemicals, Ann Arbor, Michigan, USA respectively, followed by measurement at 450 nm according to manufacturer’s instructions. For PGE_2_, standards from 39–2500 pg/ml were used and sensitivity of the assay was 36.2 pg/ml. The 8-iso-PGF_2α_ assay has a range of standards from 0.5–500 pg/ml with the sensitivity of approx. 3 pg/ml.

### Immunoblotting

Rat primary microglia were left treated with cap (1–25 µM) for 30 min then the LPS (10 ng/mL) was added for different time points (depending on the studied protein). After the experiment, cells were washed with cold PBS and lysed in the lysis buffer (42 mM Tris-HCl, 1.3% sodium dodecyl sulphate, 6.5% glycerin, 100 μM sodium orthovanadate and 2% phosphatase and protease inhibitors). Protein concentration of the samples was measured using the bicinchoninic acid (BCA) protein assay kit (Thermo Fisher Scientific, Bonn, Germany) according to the manufacturer’s instructions. For Western blotting, 10–20 µg of total protein from each sample was subjected to sodium dodecyl sulphate-polyacrylamide gel electrophoresis (SDS-PAGE) under reducing conditions. Afterward, proteins were transferred onto polyvinylidene fluoride (PVDF) membranes (Millipore, Germany). After blocking with 5% milk solution (BioRad, Munich, Germany) in tris-buffered saline (TBS) containing 0.1% Tween 20 (TBS-T), membranes were incubated with primary antibodies. Primary antibodies used were anti-COX-2 (1:500; Santa Cruz Biotechnology, Heidelberg, Germany), anti-mPGES-1 (1:6000; Agrisera, Vännas, Sweden), anti-COX-1 (1:500; Santa Cruz Biotechnology), anti-p-cPLA2/cPLA2 (1:500; Santa Cruz Biotechnology), anti-TRPV1 (1:1000; Alomone, Israel), TRPV1 antigen peptide (Alomone, Israel), anti-IκB-α (1:500; Santa Cruz Biotechnology), anti-p44/42 (1:1000; Cell Signaling Technology, Frankfurt, Germany), anti-p38 (1:1000; Cell Signaling Technology), anti-JNK (1:1000; Cell Signaling Technology), anti-phospho p44/42 (1:1000; Cell Signaling Technology), anti-phospho-p38 (1:1000; Cell Signaling Technology), anti-phospho MAPKAPK2 (1:1000; Assay Biotech), anti-phospho-JNK (1:1000; Cell Signaling Technology), and rabbit anti-actin (1:5000; Sigma Aldrich). Primary antibodies were diluted in TBS-T and 5% BSA. Membranes were incubated with the primary antibody overnight at 4 °C followed by incubation in secondary antibodies. After extensive washing (three times for 15 min each in TBS containing 0.1% Tween 20), proteins were detected with either horseradish peroxidase (HRP)-coupled anti-goat IgG (Santa Cruz Biotechnology) or anti-rabbit IgG (R&D systems, Wiesbaden-Nordenstadt, Germany) using enhanced chemiluminiscence (ECL) reagents (GE Healthcare, Freiburg, Germany). Densitometry analyses were performed using ImageJ software (NIH, USA), and β-actin control was used to confirm equal sample loading and normalization of the data.

### Real Time quantitative PCR

Quantitative real-time PCR (qPCR) was performed to determine the transcriptional regulation of COX-2, mPGES-1, and IL-1β by cap in activated microglia. For detecting TRPV1 transcripts, qPCR primers specific for rat TRPV1 were used. RNA preparation was done by using RNAspin mini RNA isolation kit (GE Healthcare, Freiburg, Germany) and for cDNA synthesis, 1 µg of total RNA was reverse transcribed using M-MLV reverse transcriptase and random hexamers (Biomers, Ulm, Germany). The synthesized cDNA was the template for the real-time PCR amplification that was carried out by the CFX96 real-time PCR detection system (Bio-Rad Laboratories, Inc.) using iQ^TM^ SYBR^TM^ Green supermix (Bio-Rad Laboratories GmbH, Munich, Germany). Specific primers were designed by using Universal probe library assay design center (Roche) and were obtained from Biomers (Ulm, Germany). Reaction conditions were 3 min at 95 °C, followed by 40 cycles of 15 s at 95 °C, 30 s at 50 °C and 45 s at 72 °C and every cycle was followed by plate reading. After that, 1 min at 95 °C, 1 min at 55 °C, followed by melt curve conditions of 65 °C, 95 °C with increment of 0.5 C for 5 s, followed by final plate reading. Glyceraldehyde 3-phosphate dehydrogenase (GAPDH) served as an internal control for sample normalization and the comparative cycle threshold Ct method was used for data quantification as described previously^78^. List of primer sequences can be seen in materials and methods section of Supplementary Information.

### Determination of COX enzymatic activity in microglia

To assess any direct inhibitory effect of cap on COX enzymatic activity, an arachidonic acid (AA) assay was performed. Briefly, for the COX (COX-1 + COX-2) activity microglial cells were plated in a 24-well plates and pre-incubated with LPS (10 ng/ml) for 24 h. Thereafter, medium was changed to a serum free media. Inhibitors were added for 15 min subsequently, AA was supplemented for the next 15 min. Supernatants were then collected for the determination of PGE_2_. For the COX-1 activity, the only difference in the protocol was that cells were not pre-treated with LPS, rest was followed same as for COX activity.

### Statistical analyses

Statistical analyses were performed using statistical software GraphPad Prism 5.0 (GraphPad software Inc., San Diego, CA, USA). Values of all experiments are represented as mean ± SE (standard error) of at least three independent experiments. Raw values were converted to percentage values and either control (untreated) or LPS was considered as 100% (depending upon the experimental conditions). Values were compared using one-way ANOVA with *post hoc* Student Newman–Keuls test (multiple comparisons) for variables with parametric distribution. The level of significance was set at ^#^p < 0.05, ^##^p < 0.01, ^###^p < 0.001 compared with control conditions (untreated) and *p < 0.05, **p < 0.01, ***p < 0.001 with respect to LPS (treated).

## Electronic supplementary material

Supplementary Information
