# Supplementary material for: Alleviation of Microglial Activation Induced by p38 MAPK/MK2/PGE2 Axis by Capsaicin: Potential Involvement of other than TRPV1 Mechanism/s
Source: Sci Rep. 2017 Mar 8;7:116. doi: 10.1038/s41598-017-00225-5 (PMC5428011; doi:10.1038/s41598-017-00225-5)
Supplement: Supplementary file 1 — Supplementary Information [file 41598_2017_225_MOESM1_ESM.pdf]

Supplementary Information

Alleviation of Microglial Activation Induced by p38 MAPK/MK2/PGE_2 Axis by Capsaicin: Potential Involvement of other than TRPV1 Mechanism/s

Harsharan S. Bhatia[1, 2*], Nora Roelofs[1], Eduardo Muñoz[3,4], Bernd L. Fiebich[1, 2]

[1]Department of Psychiatry and Psychotherapy, University of Freiburg Medical School, Hauptstrasse 5, D-79104, Freiburg, Germany

[2]VivaCell Biotechnology GmbH, Ferdinand-Porsche-Strasse 5, D-79211, Denzlingen, Germany

[3]Maimonides Biomedical Research Institute of Córdoba, Reina Sofía University Hospital, Department of Cell Biology, Physiology and Immunology, University of Córdoba, Avda Menéndez Pidal s/n., 14004, Córdoba, Spain

[4]VivaCell Biotechnology España, Parque Científico Tecnológico Rabanales 21, 14014, Córdoba, Spain

[*]Corresponding author: Harsharan S. Bhatia, Ph.D.
Department of Psychiatry
University of Freiburg Medical School
Hauptstr. 5
D - 79104 Freiburg, Germany
Tel. (49) 761/270-68980
Fax (49) 761/270-69170
E-mail: harsharan.singh.bhatia@uniklinik-freiburg.de

Materials and Methods

Viability Assays

Viability of primary microglia after treatment with capsaicin was measured by the CellTiter-Glo® luminescent cell viability assay (Promega), which is used to determine the number of metabolically active and viable cells in cell culture based on quantitation of the ATP present in the cells. Additionally, lactate dehydrogenase (LDH) assay (Roche diagnostic, GmbH) was used for microglia as well as for OHSCs. This colorimetric assay quantitatively measure LDH released into the media from damaged cells as a biomarker for cellular cytotoxicity and cytolysis. Briefly, cells (2 × 10^5/ml) or tissues (5-6 hippocampi/well) were cultured for 24 h and then incubated with cap (0.01-25 µM) for 30 min. Thereafter, cells were incubated with or without LPS for the next 24 h. DMSO (10 % end conc.) was used as positive control to validate the functionality of the assays. The conc. of ATP was measured after adding 100 µl of reconstituted substrate and incubation time of 10 min. Luminescence were measured in GloMax® Luminometer. In case of LDH assay, cell and tissue supernatants were incubated with 100 µl of freshly prepared reaction mixture (catalyst and dye solution) and incubated for 30 min at room temperature. Thereafter, measurements were performed at 490 nm with reference wavelength of 600 nm.

Measurement of TNF-α, IL-6 and IL-1β release

Effects of capsaicin were also studied by determining the release of various cytokines. Briefly, cells and OHSCs were pre-incubated with capsaicin for 30 min. Afterwards LPS was added for 24 h and release of TNF-α, IL-6 and IL-1β were determined in the supernatants which were collected after centrifugation at 1000g for 5 min at 4°C. For determination of rat TNF-α, mouse TNF-α & IL-6 (eBioscience, Frankfurt, Germany), rat IL-6 (Thermo Fisher Scientific, Darmstadt, Germany), human TNF, IL-6, IL-1β (eBioscience, Frankfurt, Germany) commercially available ELISA kits were used. All the measurements were done at 450 nm according to the manufacturer's instructions.

Immunofluorescence and Imaging

For the slice morphology and visualization of cell population of microglia and neurons in OHSC, immunofluorescent staining was performed. Briefly, after 6 DIV slices were washed briefly with warm PBS (37 °C) and fixed with 4% paraformaldehyde (PFA) solution (wt/vol in PBS) for overnight at 4 °C. For immunofluorescent staining single slice cultures on the membrane were cut with a scalpel and transferred to a 24-well plate. Prior to incubation with the primary antibody, the tissue was permeabilized and blocked using 5% normal goat serum (Vector Laboratories) in PBS containing 0.3% Triton-X-100 (Sigma-Aldrich). Incubation with the primary and secondary antibodies was performed in PBS containing 1% normal goat serum and 0.3% Triton-X-100. Each incubation step was performed over night at 4 °C followed by three washing steps for 10 min in PBS. To stain microglia cells, rabbit anti-mouse Iba1 antibody (Wako Chemicals, Neuss, Germany) in a 1:1000 dilution and secondary antibody donkey anti-rabbit IgG Alexa 647 (Life technologies) in 1:1000 dilution was used. To get an overview of the slice morphology and the hippocampal structures, neurons were stained with the mouse anti-mouse NeuN antibody (Millipore) in 1:1000 dilution, combined with the secondary antibody donkey anti-mouse IgG Alexa 488 (Life technologies). Stained slices were mounted on slides and embedded with fluorescence mounting medium (Dako, Hamburg, Germany). The immunofluorescent labeled slices were analyzed by confocal laser scanning microscopy (LSM) using a ZEISS LSM 510 META (Carl Zeiss AG, Oberkochen, Germany). Whole Images of OHSC were observed with a C-Apochromat 10 × /0.45 W objectives and for high magnification a LD LCI Plan-Apochromat 25 × /0.8 Imm Corr DIC objective was used.

Rat specific Primer Sequences

The following primer sequences were used in the present study.

COX-2: Fwd 5'-CTACACCAGGGCCTTCC-3'; Rev 5'-TCCAGAACTTCTTTTGAATCAGG-3'; **mPGES-1**: Fwd 5'-GCACACTGCTGGTCATCAAG-3'; Rev 5'-ACGTTTCAACGC

GTCCTC-3'; **IL-1β**: Fwd 5'-TGTGATGAAAGACGGCACAC-3'; Rev 5'-CTTCTTCTTTG GGTATTGTTTGG-3'; **TRPV1**: Fwd 5'-CAACAGGAAGGGGCTCAC-3'; Rev 5'-TCTGG GAATGTAGGCCAAGAC-3'; **GAPDH**: Fwd 5'-TGGGAAGCTGGTCATCAAC-3'; Rev 5'-GCATCACCCCATTTGATGTT-3'.

Figures

Fig.S1 Effects of capsaicin on the release and expression of pro-inflammatory mediators in rat microglia

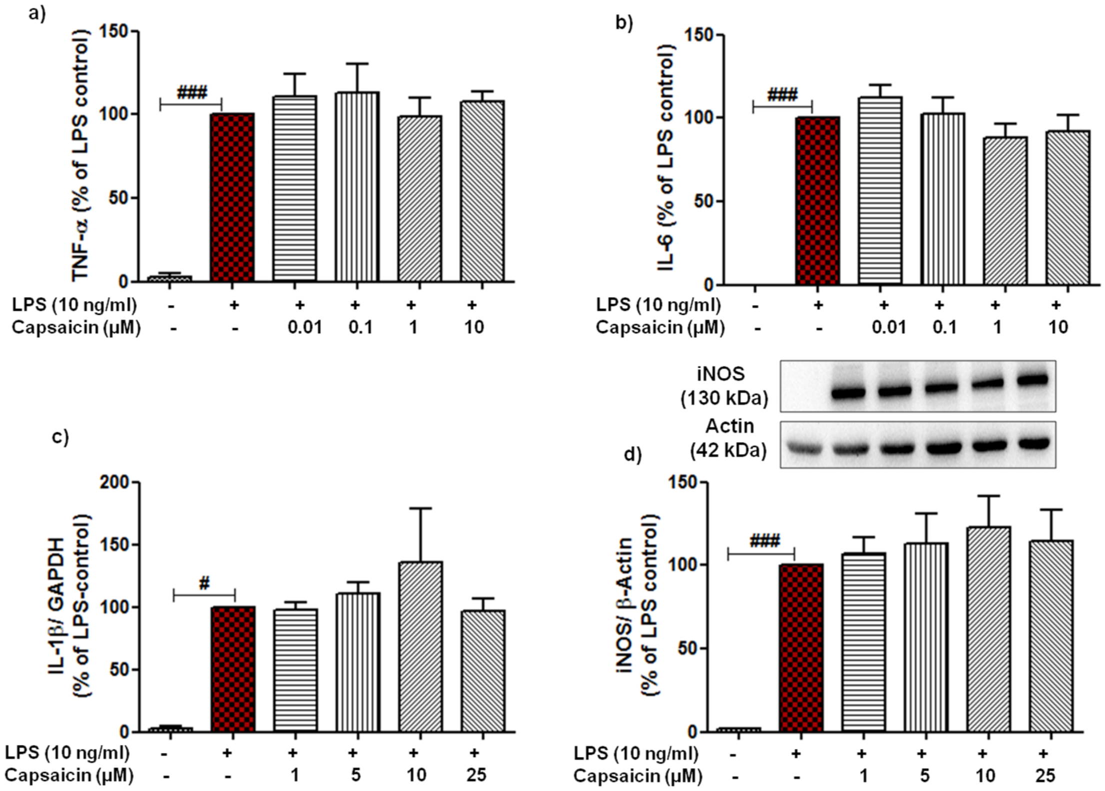

Fig.S1 Effects of capsaicin on the release and expression of pro-inflammatory mediators in rat microglia. Microglia were pre-treated with capsaicin (0.01-10 μM) for 30 min, afterwards cells were incubated with or without LPS (10 ng/ml) for 24 h. At the end of incubation, cell supernatants were collected; centrifuged and release of TNF-α (a), IL-6 (b) was quantified by using ELISA assays. For the gene expression and protein levels of inflammatory mediators, similarly cells were treated with capsaicin (1-25 μM) for 30 min, thereafter incubated with or without LPS (10 ng/ml) either for 4 h or 24 h for studying IL-1β gene expression (c) and

inducible nitric oxide (iNOS) protein levels (d). For protein, whole cell lysates were subjected to western blot for specific iNOS antibody. Representative blots are shown (d, upper panel) and densitometry analyses were performed (d, lower panel). To confirm equal sample loading, membranes were stripped and reprobed for β-actin and the data were used for normalisation. Data are presented as percentage control of LPS. Statistical analyses were carried out by using one way ANOVA with *post hoc* student Newman-Keuls test (multiple comparisons). Results are expressed as means ± SE of 3-5 independent experiments. $^{\#}p<0.001$; $^{\#\#\#}p<0.001$ compared with control cells.

Fig. S2 Effects of capsaicin on the viability of microglia

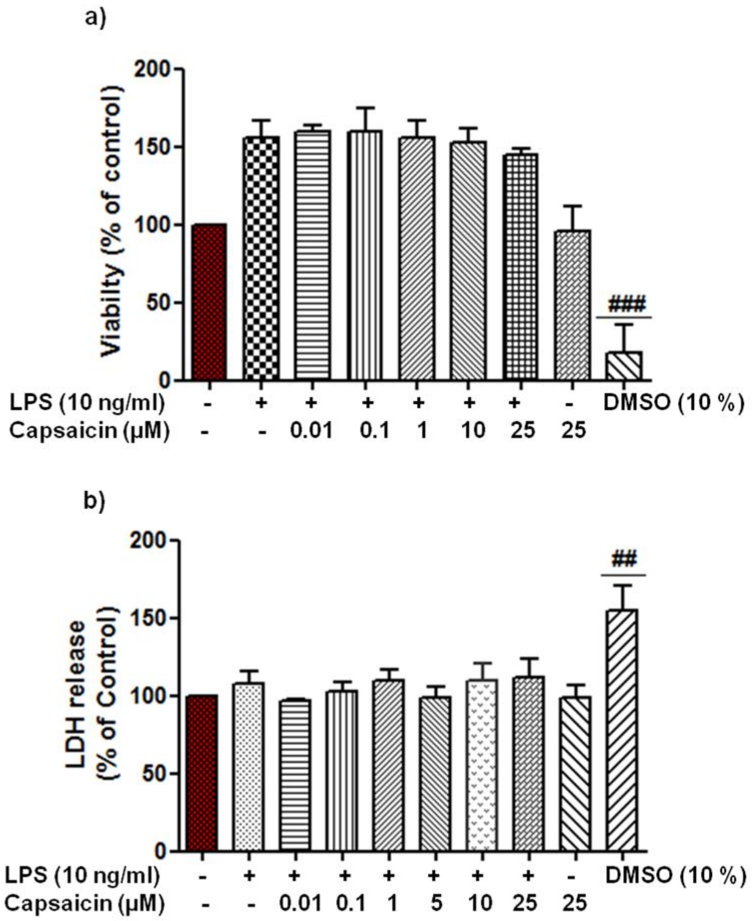

Fig. S2 Effects of capsaicin on the viability of microglia. Microglia were pre-treated with capsaicin (0.01-25 μM) for 30 min, afterwards cells were incubated with or without LPS (10 ng/ml) for the next 24 h. DMSO (10%) was used as positive control to affect cell viability

significantly. At the end of incubation, a) ATP and b) LDH assays were performed to assess the microglia cell viability. For a detailed measurement protocol, see materials and methods section. Results are expressed as means ± SE of 3 independent experiments. ##$p<0.001$; ###$p<0.001$ compared with control cells.

Fig. S3 Expression of TRPV1 in microglia

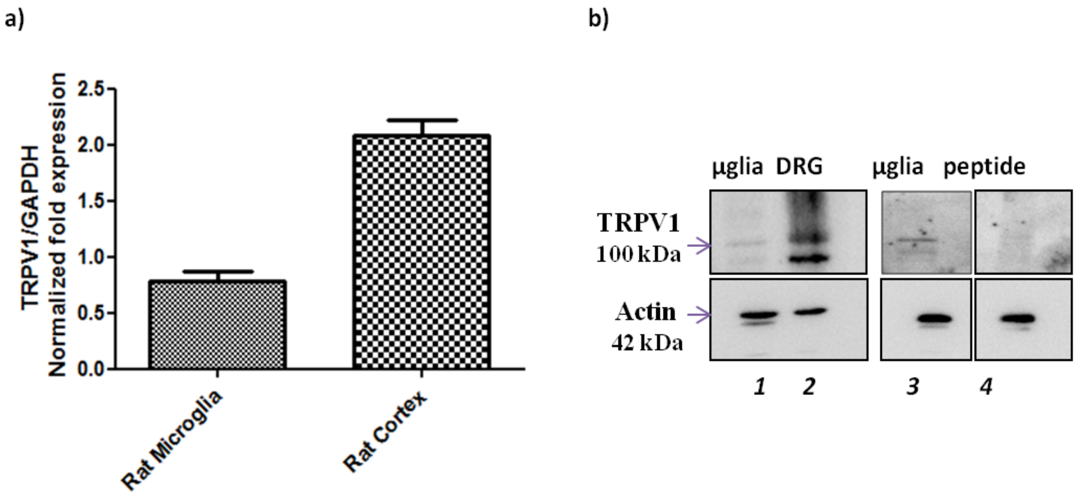

Fig. S3 Expression of TRPV1 in microglia. Primary rat microglia cells were used to analyse the expression of TRPV1 gene expression and protein levels. cDNA was prepared from control microglia cells which were subjected to real-time quantitative PCR that was carried out by the CFX96 real-time PCR detection. Rat brain cortex was used as positive control for the validation and functionality of rat specific TRPV1 primers. GAPDH was used as an internal control for normalisation (a). For the protein levels, control microglia lysates were subjected to immunoblot and stained with specific antibody for TRPV1 (b, lane1 & 3). Neonatal rat dorsal root ganglion (DRGs) lysate was used as positive control for the validity and functionality of TRPV1 antibody (b, lane 2). To confirm the specificity and right molecular weight of anti-TRPV1 antibody, control microglia lysates were pre-incubated with the control peptide antigen for 30 min at RT (b, lane 4). Thereafter lysates were incubated with anti-TRPV1 antibody and preceded similarly as described in immunoblotting sub-section

of materials and methods section. For equal sample loading, membranes were stripped and re-probed for β-actin (b, lower panel)

Fig. S4 Effects of capsaicin on the release of cytokines in LPS activated mouse microglia

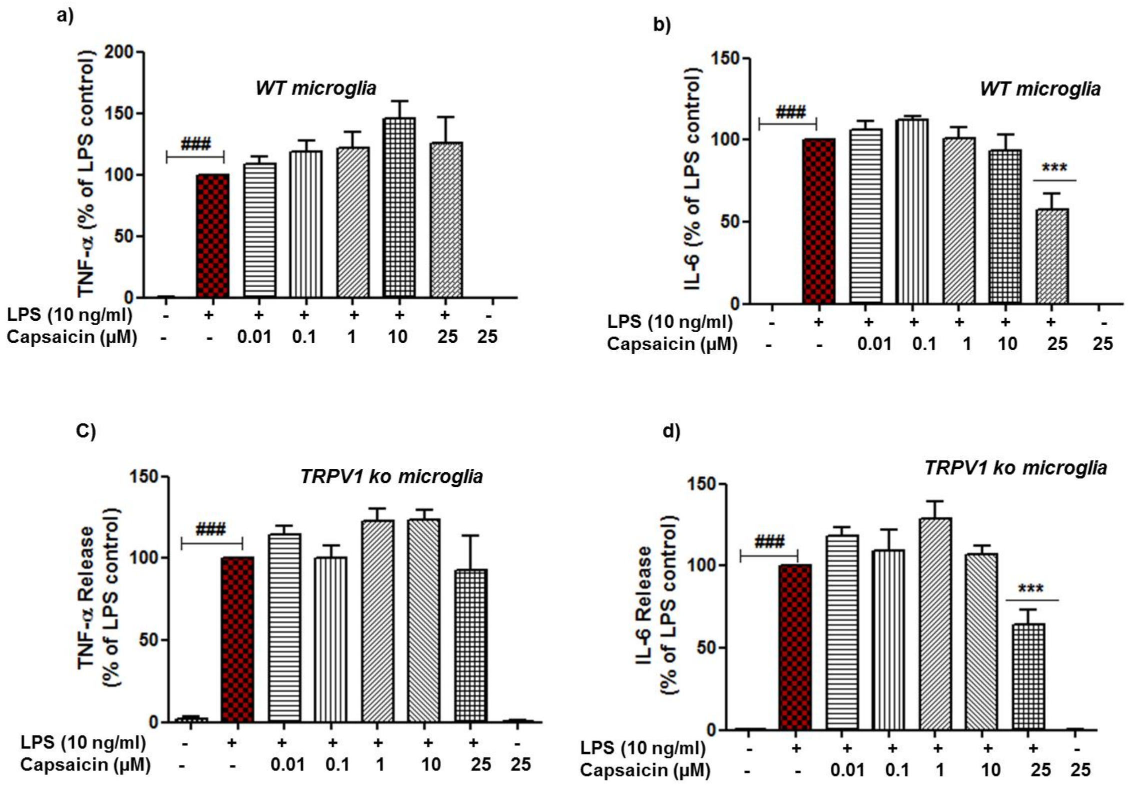

Fig. S4 Effects of capsaicin on the release of cytokines in LPS activated mouse microglia cultures prepared either from wild type (wt) or TRPV$^{-/-}$ mice. Cultures were pre-treated with capsaicin (0.01-25 μM) for 30 min thereafter incubated with or without LPS (10 ng/ml) for the next 24 h. At the end of incubation, cell supernatants were collected and release of TNF-α (a, c) and IL-6 (b, d) were measured by ELISA. Statistical analysis was carried out by using one way ANOVA with *post hoc* student Newman-Keuls test (Multiple comparisons). Results are expressed as means ± SE of three independent experiments. $^{###}p<0.001$ compared with control cells. $^{***}p<0.001$ compared with LPS activated cells.

Fig. S5 Showing slice morphology and the hippocampal structures

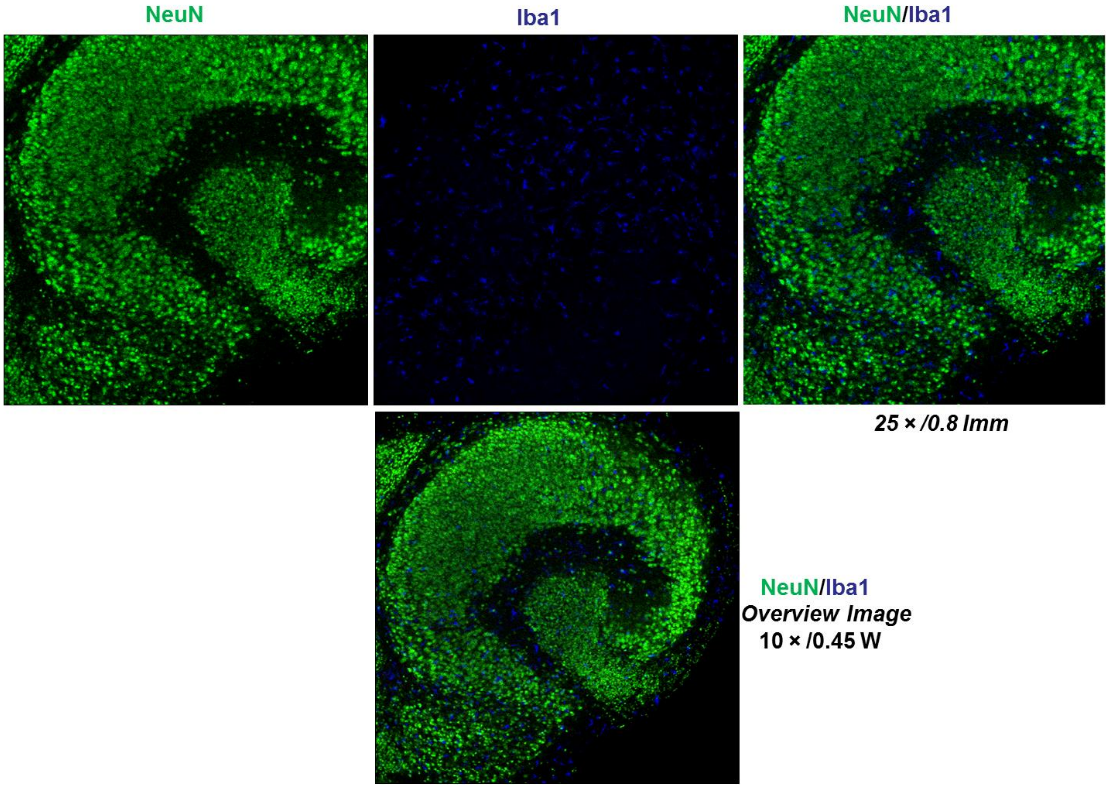

Fig. S5 Showing slice morphology and the hippocampal structures. Neurons were stained with NeuN antibody (a) microglia with Iba1 (b) merged image of NeuN/Iba1 (c). Stained slices were mounted on slides and embedded with fluorescence mounting medium. The immunofluorescent labeled slices were analyzed by confocal laser scanning microscopy (LSM) using a ZEISS LSM 510. High magnification images of OHSC were observed and taken using a LD LCI Plan-Apochromat 25 × /0.8 Imm Corr DIC objective as depicted (a-c) and whole merged image by C-Apochromat 10 × /0.45 W objective as shown (d). Scale bars: 100 µM.

Fig. S6 Effects of capsaicin on the release of TNF-α and IL-6 in OHSCs

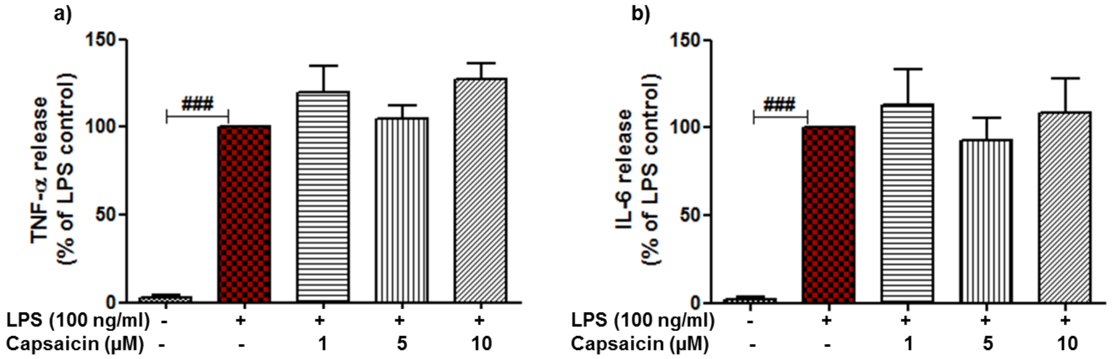

Fig. S6 Effects of capsaicin on the release of TNF-α and IL-6 in OHSCs. Slices were pre-treated with capsaicin (1-10 μM) for 30 min followed by incubation with or without LPS (100 ng/ml) for the next 24 h. At the end of incubation, cell supernatants were collected; centrifuged and release of TNF-α (a), IL-6 (b) was quantified by using mouse specific ELISA assays. Data are presented as percentage control of LPS. Statistical analyses were carried out by using one way ANOVA with *post hoc* student Newman-Keuls test (multiple comparisons). Results are expressed as means ± SE of 3 independent experiments. $^{###}p<0.001$ compared with control slices.

Fig. S7 Effects of capsaicin on the synthesis and activation of cPLA2 and levels of COX-1

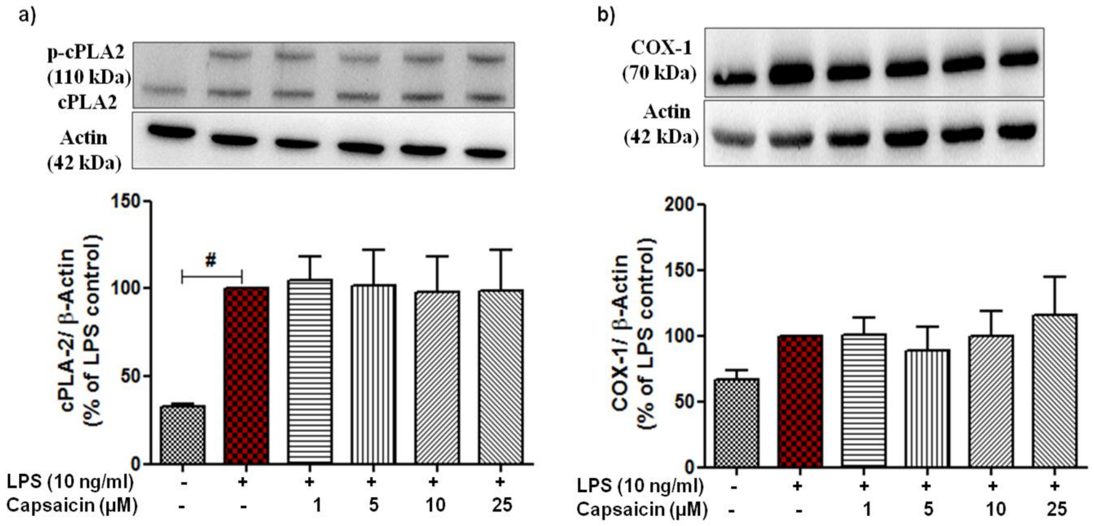

Fig.S7 Effects of capsaicin on the synthesis and activation of cPLA2 and levels of COX-1. Cells were pre-treated with capsaicin (1-25 µM) for 30 min followed by incubation of cells with or without LPS (10 ng/ml) for the next 24 h. Whole cell lysates were subjected to western blot for cPLA2 and COX-1. Representative blots for phospho cPLA2/cPLA2 and COX-1 are shown (a & b upper panel) and densitometry analyses were performed (a & b, lower panel). To confirm equal sample loading, membranes were stripped and reprobed for β-actin and the data were used for normalisation. $^{\#}p<0.05$ compared with control cells.

Fig. S8 Effects of capsaicin on COX and COX-1 enzymatic activity in microglia

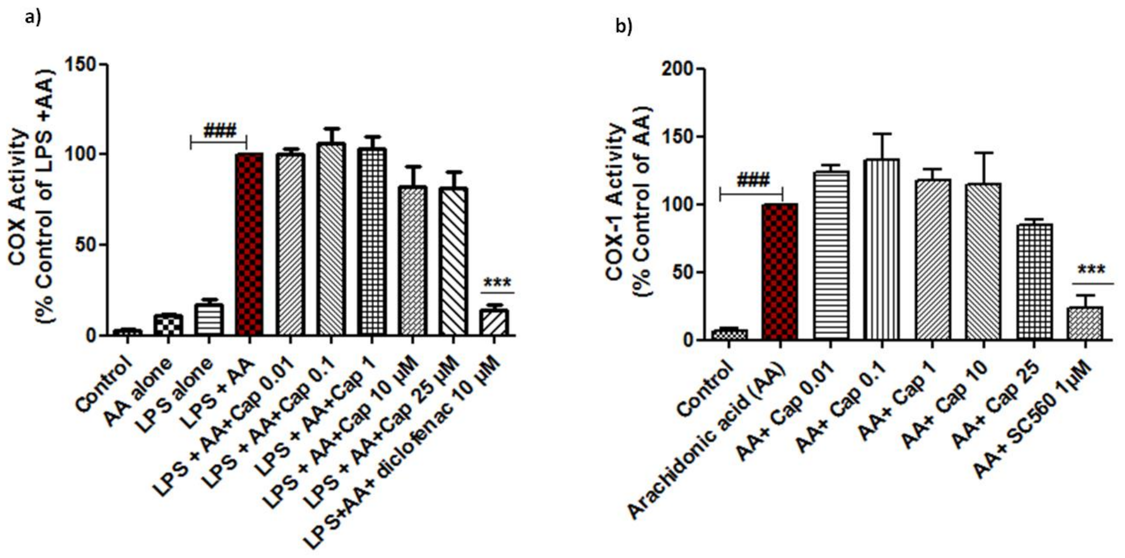

Fig. S8 Effects of capsaicin on COX and COX-1 enzymatic activity in microglia. Cells were either treated with LPS (10 ng/ml) or left untreated for the period of 24 h. Afterwards, media was removed and replaced with fresh serum free media. Thereafter cells were treated with different concentrations of capsaicin or other drugs for 30 min in absence or presence of arachidonic acid. Cell supernatants were collected and PGE_2 for a) COX and b) COX-1 activities were measured as described in materials and methods section. Statistical analyses were carried out by using one way ANOVA with *post hoc* student Newman-Keuls test (multiple comparisons). Results are expressed as means ± SE of three independent experiments. AA (15 µM) + LPS (10 ng/ml) or AA (15 µM) was taken as 100 % and

compared with either controlcells or AA (15 µM) or LPS (10 ng/ml). ###p<0.001 compared with control; ***p<0.001 with respect to AA + LPS or AA.

Fig. S9. Effects of capsaicin on the degradation of IκBα induced by LPS

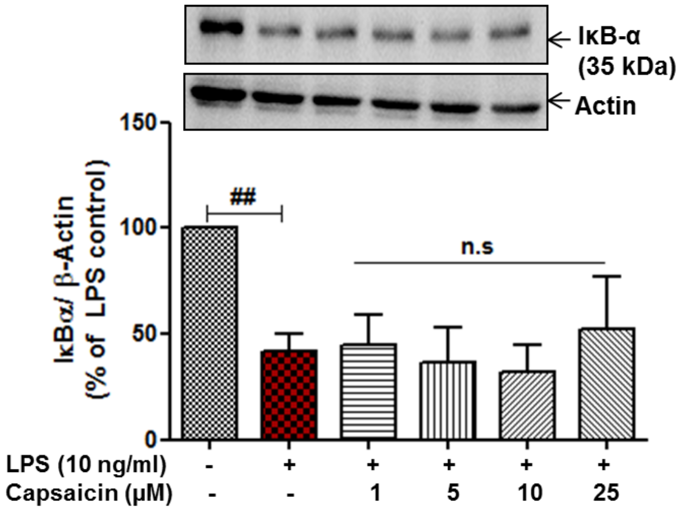

Fig.S9. Effects of capsaicin on the degradation of IκBα induced by LPS. Cells were pre-treated with capsaicin (1-25 µM) for 30 min followed by stimulation with or without LPS (10 ng/ml) for 15 min. Whole cell lysates were subjected to western blots analyses. Representative blots (upper panel) and densitometry analyses (lower panel) are shown. Statistical analyses were carried out by using one way ANOVA with *post hoc* student Newman-Keuls test (multiple comparisons). Results are expressed as means ± SE of three 3 independent experiments. ##p<0.01 compared with LPS (10 ng/ml), n.s; not significant.

Fig. S10. Showing original blots

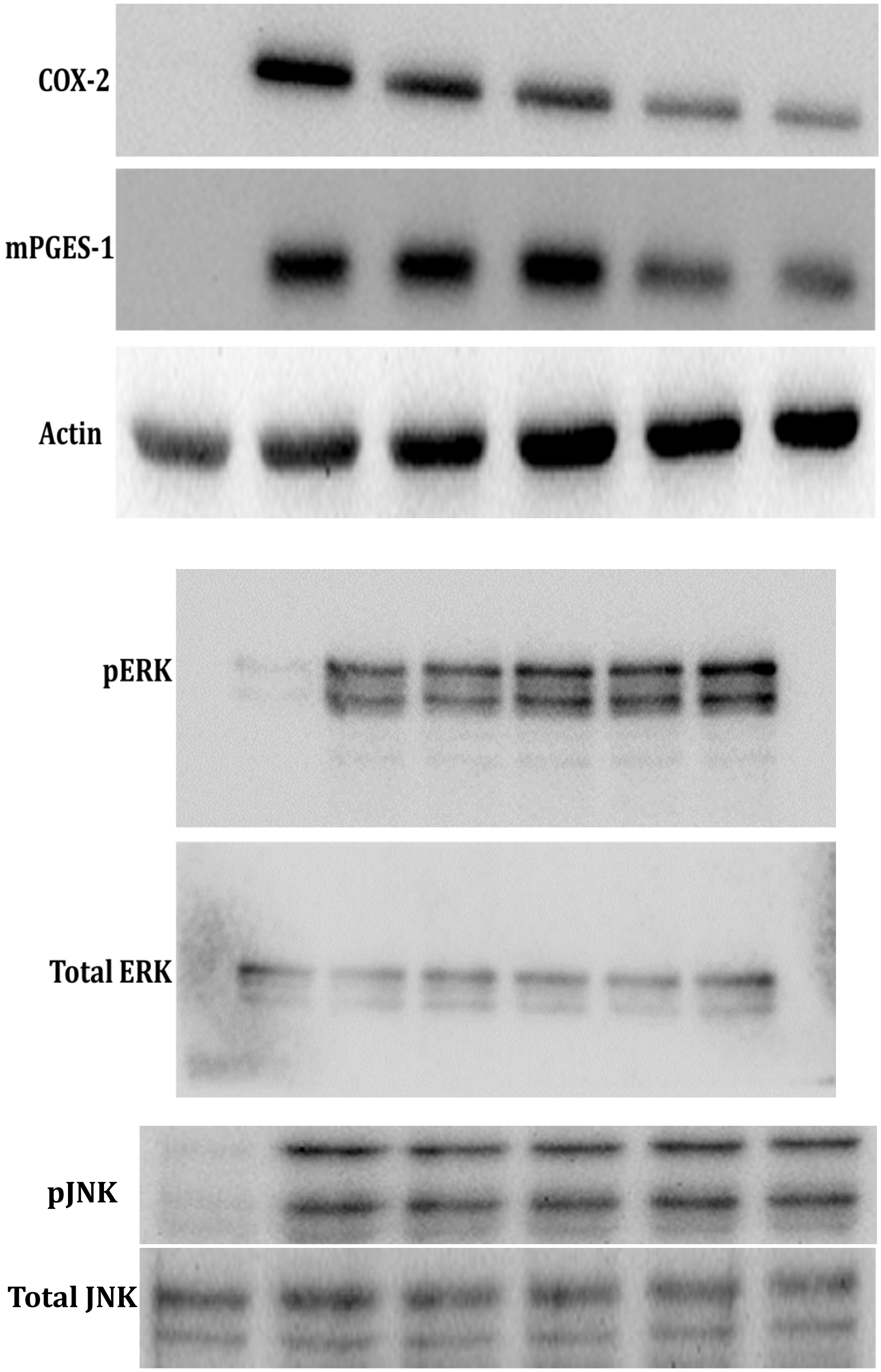

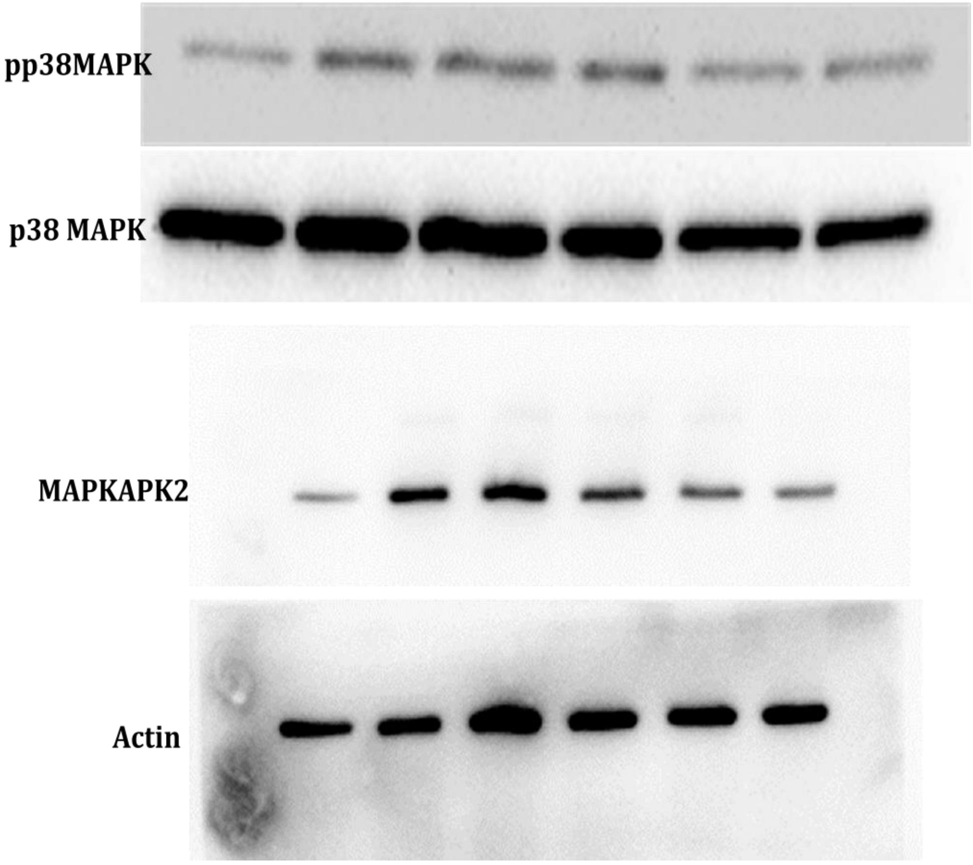

Fig. S10. Showing original blots of studied protein targets. After the successful transfer of protein on PVDF membranes, membranes were blocked and eventually cropped horizontally according to the desired protein of interest. For mPGES-1 (17 kda), Actin (42 kda) and COX-2 (72 kda), membranes were cropped at 25 kda, 35 kda, 55 kda and just below 100 kda. For kinases, membranes were cropped between 60 kda and 35 kda (depending on the desired protein) to detect ERK (44/42 kda), JNK (54/46 kda), p38 (43 kda), MAPKAPK2 (49 kda) and Actin (42 kda). Thereafter each membrane was processed for respective antibody incubation and detection as described in method section of main text.
